# Polyethylene and Polypropylene Pyrolysis Using Fe^3+^-Modified Kaolin Catalyst for Enhanced Gas and Pyrolysis Oil Production

**DOI:** 10.3390/polym17212963

**Published:** 2025-11-06

**Authors:** Sergey Nechipurenko, Binara Dossumova, Sergey Efremov, Nazar Zabara, Aigerim Kaiaidarova, Olga Ibragimova, Anara Omarova, Fedor Pogorov, Diyar Tokmurzin

**Affiliations:** 1 Center of Physical Chemical Methods of Research and Analysis, Faculty of Chemistry and Chemical Technology, Al-Farabi Kazakh National University, Almaty 050012, Kazakhstan; sergey.nechipurenko@kaznu.edu.kz (S.N.); dossumova63@mail.ru (B.D.); efremsa@mail.ru (S.E.); nzr.zabara@gmail.com (N.Z.); aigerim_ko@list.ru (A.K.); ibragimova@cfhma.kz (O.I.); omarova@cfhma.kz (A.O.); fpogorov@gmail.com (F.P.); 2 Laboratory for Chemical Technology, Ghent University, Industriele Scheikunde, Technologiepark-Zwijnaarde 125, 9052 Gent, Belgium

**Keywords:** catalytic pyrolysis, kaolin, waste plastics, thermogravimetric analysis, slow pyrolysis, polypropylene, polyethylene

## Abstract

Calcined and acid-leached kaolin impregnated with Fe(NO_3_)_3_·9H_2_O (6.6 wt. % Fe_2_O_3_) was developed as an inexpensive bifunctional catalyst for the slow fixed-bed pyrolysis of polypropylene (PP) and low-density polyethylene (LDPE). Experiments were run with catalyst-to-plastic mass ratios of 1:4, 1:2, and 1:1 in a quartz tube reactor heated from 25 to 800 °C. For PP, increasing the Fe/kaolin loading progressively raised non-condensable gas from 26 wt. % to 44 wt. % and drove liquid aromatics from 27.9% to 72.3%, while combined paraffins olefins fell to 2.5% and wax exhibited a 46 → 24 → 36 wt. % trend. In contrast, LDPE at a 1:4 ratio already yielded 56 wt. % oil and only 22 wt. % wax; further catalyst addition mainly enhanced CH_4_/CO-rich pyrolysis gas (PyGas) and char without substantially boosting aromatics. Gas analysis confirmed that Fe_2_O_3_ reduction and kaolin de-hydroxylation generated in situ H_2_O, CO, and H_2_. Given the catalyst’s low cost, regenerability, and ability to valorize the two most abundant waste polyolefins within the same reactor, the process offers a scalable route to flexible fuel and gas production from mixed plastic streams.

## 1. Introduction

**Figure 1:**
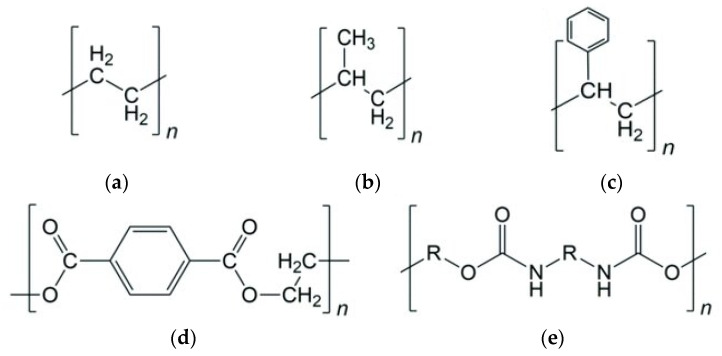
Chemical structure of common plastics: (**a**) polyethylene, (**b**) polypropylene, (**c**) polystyrene, (**d**) polyethylene terephthalate, (**e**) polyurethane (R = -Ph-CH2-Ph-).

Plastic waste accumulation has become a critical environmental challenge, motivating research in chemical recycling technologies that convert waste polymers into fuels and chemicals: over 380 Mt/y of plastic waste was produced in 2017. According to 2023 statistics, pyrolysis became the most popular method of plastic chemical recycling due to its economic characteristics and the value of products produced [1]. Pyrolysis—the thermal decomposition of polymers in an inert atmosphere—is a promising route for producing liquid hydrocarbon fuels from plastics. Figure 1 describes the chemical structure of common polymers, such as PE, PP, PS, PET, and PUR. While PE and PP comprise linear polyolefin polymer structure, PET, PS, and PUR have phenyl elements in their structure. PET and PUR also have oxygen in their structure. Conventional pyrolysis of polyolefins like polyethylene (PE) and polypropylene (PP) typically requires high temperatures (500–800 °C) and yields a broad product distribution of gases, oil, wax, and char [2]. Pyrolysis of PS, PET, and PUR produces gas, char, and oil mostly consisting of aromatics instead of wax [3,4,5]. The use of catalysts in the pyrolysis process (catalytic pyrolysis) can significantly improve the yield and quality of liquid products while reducing the required reaction temperature. In particular, solid acid catalysts promote cracking of long polymer chains into lighter hydrocarbons and dehydrogenation/cyclization reactions that form aromatic compounds, thereby increasing the gasoline-range content of the oil [6].

Among potential catalysts, kaolin-based catalysts have gained attention due to their low cost, natural abundance, and moderate acidity. Kaolin is a clay mineral primarily composed of kaolinite (Al_2_Si_2_O_5_(OH)_4_) with a layered silicate structure. In its raw form, kaolin has a relatively low surface area and mild Brønsted/Lewis acidity, which tends to favor the production of heavier liquid hydrocarbons rather than light gases [7]. This contrasts with strongly acidic zeolite catalysts (e.g., H-ZSM-5) that often over-crack polymers into light gases and coke due to micropore shape-selectivity and high acid density. Kaolin’s milder acidity can thus be advantageous for maximizing liquid oil yield in pyrolysis. Moreover, kaolin and related clays can be activated or modified (for instance by acid treatment or ion exchange) to tune their surface area and acidity. Such modifications have been shown to enhance catalytic performance, bridging the activity gap between raw clay and synthetic zeolites.

Untreated (or “neat”) kaolin powder has been used as a baseline catalyst in many studies. Raw kaolin typically has a low BET surface area (5–20 m^2^/g) and contains both Brønsted (from surface –OH groups) and Lewis acid sites associated with aluminum in the clay structure. Despite its moderate acidity, raw kaolin can already catalyze polymer cracking to some extent. For instance, Eldahshory et al. found that natural kaolin (without any modification) improved oil yield from PP by ~10 wt. % compared to thermal pyrolysis [8]. Similarly, Kumar et al. observed that adding 10–20% raw kaolin to LDPE in a batch pyrolyzer not only increased liquid yield but also lowered the apparent activation energy of decomposition by ~15%, indicating catalytic facilitation of C–C bond breaking [9]. To enhance kaolin’s performance, researchers have employed acid modifications. Acid treatments (using HCl, H_2_SO_4_, HNO_3_, etc.) leach out some octahedral Al and impurities from kaolin, often increasing its surface area and creating additional mesoporosity and acid sites [10]. Luo et al. treated kaolin with HCl and observed a marked increase in catalytic activity for PP pyrolysis [7]. In their 2020 study, HCl-modified kaolin gave an oil yield of up to 90–97% (almost complete conversion of PP to liquids and gases) versus substantially lower yields with untreated clay. The acid-treated kaolin produced predominantly liquid hydrocarbons in the gasoline boiling range, with a high aromatic content [11]. This is attributed to stronger acid sites generated by leaching (possibly exposing more Si-OH (Brønsted) groups and structural defects acting as Lewis acids). Panda et al. (2014) likewise reported that acid-activated kaolin significantly improved PP conversion to oil, achieving ~92% liquid yield in one case [12]. However, over-zealous acid treatment can also collapse the clay structure; thus, an optimal acid concentration/time is usually sought to maximize surface area without destroying the layered framework.

Luo et al. explored the performance of low-cost kaolin as a catalyst for the catalytic pyrolysis of low-density polyethylene (LDPE), focusing on how particle size influences product distribution and how the catalyst’s properties evolve during repeated use [13]. LDPE granules with a carbon content of 85.2 wt. % were pyrolyzed at 600 °C under nitrogen, using kaolin powders of three sizes—325 mesh (K325), 800 mesh (K800), and 1250 mesh (K1250)—at a catalyst-to-plastic mass ratio of 1:9. The results showed that all kaolin catalysts produced liquids rich in aliphatics and aromatics, with hydrocarbons in the C_6_–C_20_ range making up 90–95% of the liquid phase. The first use of K1250 yielded the highest proportion of aliphatics (64.66%) and total hydrocarbons (93.91%), while also producing a significant amount of hydrogen in the gas phase (30.07%). Smaller particle sizes generally promoted the cracking of heavier fractions into lighter aliphatics and gases, and gas yields increased both with decreasing particle size and with catalyst reuse—K1250-3, for example, gave 31.6 wt. % gas compared to 23.9 wt. % without a catalyst.

Li et al. focused on developing efficient, low-cost catalysts for converting mixed plastic waste into valuable fuels through catalytic pyrolysis. The researchers selected montmorillonite-based bentonite as the starting material and modified it by pillaring with aluminum, iron, titanium, or zirconium compounds. This intercalation and calcination process expanded the interlayer spacing, increased the surface area, and adjusted the acidity of the resulting pillared clays (PILCs), creating materials with tunable textural and catalytic properties. The study proposes two main reaction pathways for the catalytic pyrolysis process, emphasizing the role of iron sites in facilitating dehydrogenation and selective cracking. Overall, the work demonstrates that Fe-PILC is a promising, scalable catalyst for upgrading mixed plastic waste into high-value fuels such as diesel and hydrogen, outperforming traditional Al-PILC in both selectivity and yield.

Kumar et al. (2024) examined the influence of kaolin catalyst on the pyrolysis of low-density polyethylene (LDPE) waste in a batch reactor at 550–650 °C and heating rates of 5–25 °C min^−1^ [9,14]. Kaolin loadings of 10–20 wt. % were compared with thermal pyrolysis, with reaction kinetics evaluated via thermogravimetric analysis. The catalyst reduced the decomposition temperature by 10–20 °C and lowered the apparent activation energy from 162–166 kJ mol^−1^ to 138–145 kJ mol^−1^. Optimal conditions (600 °C, 20 °C min^−1^, 20 wt. % kaolin) maximized pyrolytic oil yield while minimizing char formation. Catalytic treatment produced lighter, less viscous oils with higher aromatic content, as confirmed by FTIR, and generated gases including hydrogen, propane, butane, and propylene (GC analysis). These findings demonstrate that kaolin can enhance LDPE pyrolysis efficiency, improve liquid fuel quality, and reduce the energy barrier, highlighting its potential as a low-cost, effective catalyst for converting plastic waste into cleaner fuels.

Eldahshory et al. (2023) investigated the catalytic pyrolysis of waste polypropylene (WPP) using three low-cost Egyptian natural minerals—kaolin, hematite, and white sand—at varying catalyst-to-plastic ratios (1:1–1:8) [8]. The catalysts were characterized by XRF and BET analyses, while WPP degradation behavior was assessed via TGA; the resulting oils were analyzed using GC–MS and DSC. Non-catalytic pyrolysis at 500 °C yielded approximately 70 wt. % oil. Among the catalysts, kaolin at a 1:2 ratio achieved the highest oil yield (80.75 wt. %), outperforming hematite (70 wt. %) and white sand (68 wt. %), while white sand at 1:8 produced the highest gas yield (44 wt. %). Kaolin also generated oil with the lowest heavy fraction (25.98%) and highest light fraction (25.37%) and exhibited the lowest production cost (0.28 USD kg^−1^). The findings highlight kaolin’s superior catalytic performance and economic advantage, demonstrating its potential to enhance both yield and quality in WPP pyrolysis compared with other natural catalysts and non-catalytic operation.

Increasing the catalyst-to-plastic ratio generally promotes deeper cracking of polyethylene (PE) and polypropylene (PP), shifting the product distribution towards lighter fractions. For example, using kaolin for PP pyrolysis, a 1:2 catalyst-to-plastic ratio gave the highest oil yield (~80.8 wt. % liquid) [8]. Raising the ratio to 1:1 (more catalyst) caused over-cracking, slightly reducing oil yield (~78.3%) and increasing gas production. Similarly, dropping the kaolin catalyst loading to 1:4, 1:6, and 1:8 reduced the oil yield from waste polypropylene. Eldahshory et al. also noted the highest percentage of light oils and the lowest percentage of heavy oils at a 1:2 ratio. The use of a 1:10 catalyst-to-waste-tire ratio when iron-impregnated kaolin and bentonite were used demonstrated higher PyOil yield than non-catalytic experiments and catalytic experiments with unmodified and nitric acid-washed kaolin and bentonite [15].

The past decade of research has established kaolin-based catalysts as effective and economically attractive option for the catalytic pyrolysis conversion of polyethylene and polypropylene into liquid fuels. Lab-scale experiments have consistently shown that adding kaolin (in various forms) to the pyrolysis process increases polymer conversion and boosts liquid oil yields while often lowering the required reaction temperature. Kaolin’s moderate acidity and porosity steer the product slate towards longer-chain hydrocarbons in the gasoline and diesel range, distinguishing it from more acidic zeolite catalysts that favor gas and coke. Raw kaolin itself can improve yields (e.g., ~10–15% increase in oil yield for PP and PE vs. thermal cracking). The oils obtained with kaolin catalysts are rich in hydrocarbons suitable for fuels, containing mixtures of paraffins, olefins, and aromatics with low amounts of residual heavy wax or oxygenates [14]. Importantly, kaolin-based catalysts have demonstrated stability and reusability, which are critical for practical applications. They can withstand the high temperatures of pyrolysis and can be regenerated by burning off coke, with only gradual changes in activity/selectivity observed over multiple cycles [13]. Unlike expensive synthetic catalysts, natural kaolin is inexpensive and available in large quantities, making it feasible to use in bulk and even to discard and replace if fouled, without exorbitant cost. This aligns well with scaling up to industrial pyrolysis plants, where catalyst cost and turnover are key considerations. Pilot-scale trials have begun to validate kaolin catalysts in continuous operations—for example, using pelletized clay in a fluidized-bed reactor to avoid pressure drop issues [16]. The success of these trials, along with engine tests of the resulting oils, shows that kaolin-catalyzed pyrolysis oils can meet fuel specifications and that the process can be engineered for larger throughputs. In conclusion, kaolin-based catalysts provide a promising route to sustainable fuel production from PE and PP wastes, combining effectiveness with economic viability.

All previous studies with kaolin as a catalyst used raw or acid-activated kaolin (no redox metal) and typically studied one polymer at a time. Iron on clay appears mainly as Fe-pillared montmorillonite (not kaolin) and for mixed-plastic feeds. In this study we develop a low-cost, bifunctional Fe(III)-impregnated kaolin via sequential calcination, acid leaching, and Fe-nitrate impregnation and deploy it for in situ fixed-bed catalytic slow pyrolysis of PP and LDPE under one harmonized protocol. The predicted mechanism for this bifunctional catalyst, based on previous studies, consists of two processes (functions): acidic sites cause carbocationic mechanism of cracking long-chain molecules, and metal sites provide hydrogenation/dehydrogenation. Compared with the classic mechanism for thermal pyrolysis (which is a radical mechanism), the mechanism of bifunctional catalysts is more beneficial in an energy sense, which has been proven by extensive studies of the thermochemical processes [17]. The catalytic mechanism consists of the following steps: dehydrogenation (metal site), proton addition from acidic sites, cleavage (β-scission), and hydrogenation [18,19]. There is strong aromatization for PP versus a gas-rich shift with limited aromatization for LDPE, with these outcomes tied to acid–redox cooperation evidenced by in situ H_2_/CO formation.

## 2. Materials and Methods

### 2.1. Sample Preparation

The catalyst preparation procedure is schematically summarized in Figure S1 (see Supplementary Materials). The raw catalytic material, kaolin (Kyshtym kaolin, Kyshtym, Russian Federation), was milled and sieved through a 1 mm sieve. The sieved material then was calcined in a muffle furnace under an argon atmosphere at 800 °C for 3 h to induce a phase transformation from α-kaolin to γ-kaolin by altering the crystal lattice structure. Then, kaolin was leached using 3M HCl: (LenReaktiv JSC, Saint Petersburg, Russian Federation) calcined kaolin was added in a ratio of 1:20 to the acid and was stirred for 5 h at 80 °C. After completion of the acid treatment, the resulting suspension was cooled to room temperature and filtered using a Büchner funnel and a water-jet vacuum pump with white ribbon filter paper. The residue was washed using distilled water until the complete removal of residual hydrochloric acid. The washed solid was then dried in a laboratory drying oven at 105 °C for 3 h until all moisture was removed. Then the acid-leached kaolin was impregnated using Iron (III) nitrate nonahydrate (Sigma-Aldrich Chemie GmbH, Steinheim, Germany). Per 10 gof kaolin, 7.21 g of Fe(NO_3_)_3_ * 9H_2_O was dissolved in 50 mL of isopropyl alcohol and mixed for 30 min; then, 10 g. of acid-leached kaolin was added to the mixture and mixed further for 5 h at 80 °C until the formation of slurry [20,21]. The slurry was then dried in air for 12 h at 105 °C. After drying the sample was calcined in a muffle furnace in air for 2 h at 800 °C. After drying, catalyst samples were ground down to a powder. As a result, four catalytic materials were prepared: as-received kaolin, calcined kaolin, acid-leached kaolin, and kaolin impregnated with Fe(III).

### 2.2. Characterization

The plastic samples included polypropylene and polyethylene samples. Plastic samples were ground using a rotor-blade mill with a built-in sieve of 1 mm in size. Plastic samples were characterized using the CHNS/O analyzer (Vario MicroCube, Elementar Analysensysteme GmbH, Hanau, Germany). Catalytic material samples were analyzed using XRD, XRF, and BET analyses. XRF analyses were used to investigate the elemental composition of catalytic materials. XRF analyses were carried out using an Axios 1 kW wavelength-dispersive X-ray fluorescence spectrometer (Malvern Panalytical B.V., Almelo, Netherlands). The data was processed and interpreted using SuperQ (Malvern Panalytical B.V., Almelo, Netherlands) software. The error in the semi-quantitative analysis is ±20% (relative). XRD analyses were used to investigate mineral compounds contained in kaolin and their change after calcination, acid leaching, and impregnation. XRD analyses were carried out using the Bruker d8 advance (Bruker AXS SE, Karlsruhe, Germany). The porous structure was analyzed using the nitrogen adsorption technique with a Quantachrome’s Autosorb iQ (Quantachrome Instruments, Boynton Beach, FL, USA) gas sorption analyzer at 77 K. Initially, the compound was stabilized under a dynamic vacuum at 150 °C for 6 h. The nitrogen adsorption−desorption isotherms were measured within the range of relative pressures from 10^−6^ to 0.995. The specific surface area was calculated from the data obtained using the conventional BET and DFT models.

### 2.3. Thermogravimetric Analysis (TGA)

The thermal degradation characteristics of plastic samples mixed with catalysts were conducted using TGA (Netzsch STA 449, Selb, Germany). Samples were heated with 10 °C/min heating rate to 850 °C in nitrogen atmosphere with a gas flowrate of 50 mL·min^−1^. This temperature is well above the full decomposition temperature of PE and PP samples. The mass of all samples was between 40 and 70 mg, and the catalyst–plastic ratio was 1:1, 1:2, 1:4 for every catalyst–plastic combination.

### 2.4. Fixed-Bed Reactor Setup

**Figure 2:**
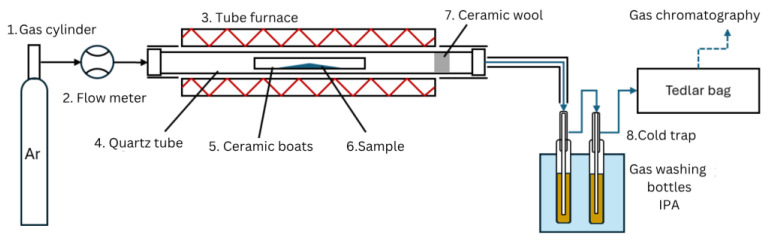
Fixed-bed reactor setup used for the slow pyrolysis of samples.

There are two main modes of catalyst-to-plastic interaction, ex situ and in situ. Ex situ mode systems typically includes two zones with plastics pyrolysis and downstream catalytic treatment of pyrolysis vapors [22]. When an in situ system is used, a mix of a plastic and a catalyst samples are catalytically pyrolyzed in a single catalytic pyrolysis zone [13,23,24]. Both ex situ and in situ reactors can be deployed using both horizontal and vertical furnaces [23,25,26]. This study uses a horizontal tube furnace with fixed bed in situ catalyst–plastic interaction mode and slow pyrolysis. The fixed-bed reactor used in the experiments is shown in Figure 2. The carrier gas (Ar) (1) is supplied from a gas cylinder, passed through the flow meter (2), and injected into the reactor inlet. The reactor (3) consisted of an externally heated quartz tube (4), where ceramic a boat (with length of 10 cm and width 1 cm) (5) with a sample (6) was placed in the middle followed by a piece of glass wool (7), preventing sample fragments entrainment with the carrier gas. Then the quartz tube (with length 1 m and diameter 60 mm) was installed inside the tube furnace. In order to improve the reactor temperature control, the ends of the quartz tube that were not covered by the furnace were thermally insulated. Additionally, to avoid liquid product condensation in the outlet tubes, the tube was also thermally insulated. The outlet of the reactor was connected to a downstream cold trap (8) for capturing the condensable products with stationary water cooling. The cold trap consisted of two impinger bottles filled with 50 mL of isopropyl alcohol and placed in the cold-water bath. After passing through the cold trap, the non-condensable gas products were collected using a Tedlar bag. The gas from Tedlar bags and the liquid fraction from the cold trap were then analyzed.

### 2.5. Experimental Procedure

Fe(NO_3_)_3_·9H_2_O-impregnated kaolin (K-Im) material and plastic samples were mixed in ratios of 1:4 (K-Im/LDPE 1:4 and K-Im/PP 1:4), 1:2 (K-Im/LDPE 1:2 and K-Im/PP 1:2), and 1:1 (K-Im/LDPE 1:1 and K-Im/PP 1:1), producing samples of 10 g each. The samples were loaded into ceramic boats and placed inside the quartz tube reactor. Argon flow was initiated at the start of the experiment and maintained throughout the entire pyrolysis process. The carrier gas (Ar) flowrate was regulated via a needle valve and injected into the tubular reactor at a 0.12 L·min^−1^ rate.

The heating program for the pyrolysis process consisted of four stages:

(1) Heating from 25 °C to 300 °C over 28 min (heating rate: 9.8 °C/min);
(2) Isothermal hold at 300 °C for 5 min, during which the gas collection bag was connected to capture the evolving volatile products and was kept open until the end of all four stages of the heating program;
(3) Heating from 300 °C to 800 °C over 50 min (heating rate: 10 °C/min);
(4) Isothermal hold at 800 °C for 15 min.

After the completion of the heating program, the furnace was turned off to cool to ambient temperature. The gas collection bag and impinger bottles were disconnected at the end of the heating program. The liquid contents from both impinger bottles were combined into a single flask and labeled accordingly. Solid residue, containing mainly charred catalyst, which remained in the reactor, was gathered when the reactor tube was cooled to ambient temperature. The solid residue was then collected, and pyrolysis wax residue was dissolved using isopropyl alcohol and separated using filtration. The formation of pyrolysis wax was caused by cold zones of the reactor near the outlet.

### 2.6. Product Collection and Analysis

**Figure 3:**
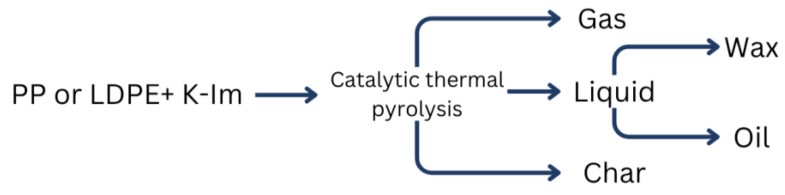
Mass balance structure of LDPE and PP.

Figure 3 schematically describes the structure of LDPE and PP catalytic pyrolysis mass balances. Catalytic thermal pyrolysis of LDPE and PP yields mainly non-condensable gas, solid residue, and condensable liquid products. Liquid products can also be further categorized as wax and oil. While wax consists of mainly long-chain hydrocarbons which solidify upon condensation, oil consists of mainly aromatic hydrocarbons and short chain hydrocarbons that remain liquid. The solid residue was collected into pre-weighed zip-lock bags and weighed using an analytical balance with a precision of 0.0001 g. The liquid phase was obtained by passing vapor products through isopropanol, which were then collected into a single sample flask. Any wax-like deposit that formed on the inner walls of the quartz reactor tube was scraped off, weighed, and then dissolved into the collected hydrocarbon mixture (liquid fraction) using a magnetic stirrer for approximately 30 min. The resulting solution was filtered using blue ribbon filter paper, and then the residue was weighed and included into the material balance. A portion of the final liquid product (approximately 5–10 mL) was sampled for subsequent analysis via GC-MS. The remaining liquid was weighed and then evaporated in a drying oven at 86 °C until a constant weight was achieved. The final residue mass was also recorded for the material balance. The Tedlar bags with non-condensable volatile products were analyzed using gas-chromatographic analysis. The total gas volume was determined using Archimedes’ principle—by measuring the volume of water displaced from a container, the corresponding volume of evolved gases was calculated.

### 2.7. GC-MS Analysis of the Isopropanol-Trapped Fraction

Before analyses, the fractions trapped in isopropanol were filtered using 0.22 µm membrane filters. Isopropanol-trapped fractions were analyzed using gas chromatography coupled with mass spectrometry (GC-MS) (Agilent 7890A/5975C, Wilmington, DE, USA). Separation was conducted using a 60 m × 0.25 mm DB-624 Ultra Inert capillary column with a 1.4 μm film thickness (Agilent, USA), operated at a 1.0 mL/min constant helium flow rate. An amount of 0.5 µL of each sample was injected into the GC inlet at 240 °C in split mode (5:1). The GC oven was heated to 40 °C and held for 5 min, then heated to 240 °C at the heating rate of 10 °C/min and held for 15 min at 240 °C), resulting in a total run time of 40 min. MS detection was performed in scan mode over a mass range *m/z* 10–550. The ion source, quadrupole, and interface temperatures of the MS were maintained at 230 °C, 150 °C, and 250 °C, respectively. MS detection was turned off from 7 to 10 min to prevent chromatographic interference from isopropanol. An amount of 500 uL of the gaseous products from the 15 L Tedlar bags was injected into GC-TCD (Agilent, USA). Analyses were conducted using two separate methods. The GC-TCD was fitted with Carboxen^®^ 1010 PLOT capillary column (30 m × 0.53 mm, 30 μm average film thickness; Supelco, USA) for the determination of hydrogen and light hydrocarbons, including CO, CH_4_, CO_2_, C_2_H_2_, C_2_H_4_, C_2_H_6_, C_3_H_6_, and C_3_H_8_. The injector temperature was set to 200 °C, with a split ratio of 2:1 for H_2_ and 50:1 for hydrocarbons. The TCD was operated at 230 °C, with a reference flow of 18 mL/min and a make-up flow of 7 mL/min. Helium was used as a carrier gas for analysis of hydrocarbons, while N_2_ was used for hydrogen. For hydrocarbons analysis, the GC oven temperature was programmed to heat to 35 °C and hold for 5 min, then to heat to 240 °C at a rate of 24 °C/min and hold for 23 min, with a total run time of 36.5 min. For hydrogen analysis the oven was programmed to heat to 35 °C and to hold the temperature for 7 min, then to heat to 240 °C at a heating rate of 60 °C/min, amounting to a total run time of 10.4 min. During the hydrogen analyses, the TCD was operated in negative polarity mode between 1 and 6.5 min. Calibration of TCD was performed using certified-standard gas mixtures, hydrogen (10% *v/v* in N_2_) and hydrocarbons (10% *v/v* of each analyte and 20% N_2_), supplied by LLC Monitoring (St. Petersburg, Russia). Certification errors for the gas mixtures ranged from 2.5% to 3% per analyte. Injection of standard gases was carried out using a 4-port valve with a 500 uL sampling loop and various split ratios.

## 3. Results and Discussion

### 3.1. PE and PP Characterization

Table 1 summarizes the CHNSO analyses of PP and LDPE used in this study. As can be seen from the table, both PP and LDPE almost exclusively comprise carbon and hydrogen, reflecting their polymer structure.

### 3.2. XRF, XRD, and BET Analysis Results

The detailed XRF analysis results are provided in the Supplementary Materials, Table S1. Most of the as-received kaolin, calcined kaolin, acid-leached kaolin, and impregnated kaolin weight comprised oxygen, 60.8 wt. %, 52.63 wt. %, 50.15 wt. %, and 48.90 wt. %, respectively. A high oxygen content is an expected analysis result because kaolinite, the main component of kaolin, is an aluminosilicate compound (Al_2_Si_2_O_5_(OH)_4_, see Table S2 in Supplementary Materials). Calcination reduced the oxygen content of the sample, suggesting kaolinite conversion to metakaolin. Table 2 summarizes the Al, Si, and Fe content of the kaolin-based samples. Figure S2 (see Supplementary Materials) demonstrates the XRD spectra of kaolin, calcined kaolin, acid-leached calcined kaolin, and iron-impregnated kaolin. Table S2 summarizes the crystalline constituents of the studied kaolin-based materials. XRD analyses demonstrated the conversion of kaolinite to a range of various crystalline alumosilicates, including alumina (ɣ-Al_2_O_3_), pyrophyllite Al_2_(Si_4_O_10_)O, silica (SiO_2_), hercynite Fe(Al_2_O_4_), and quartz (SiO_2_). Acid leaching substantially increased quartz and hercynite contents, while other crystalline compounds’ contents shrank. The high ɣ-Al_2_O_3_ content of calcined kaolin suggests that it can also be used for plasma-catalytic processes, where ɣ-Al_2_O_3_ is considered one of the favorable catalyst support materials for the packed-bed dielectric barrier discharge non-thermal plasma process [27,28,29,30]. Impregnation of kaolin with Fe(NO_3_)_3_ · 9H_2_O led to the formation of Fe_2_O_3_ on the samples. Previously, a similar process of impregnating pure alumosilicate Al_2_O_3_ with Fe(NO_3_)_3_ · 9H_2_O demonstrated that Fe_2_O_3_ was formed on the samples [31]. Table 3 describes the surface characteristics analyses of the kaolin and kaolin derivatives used in this study. Untreated kaolin rarely exceeds 5–25 m^2^ g^−1^ because its plate-like crystalloids stack densely [32,33,34]. Raw clays often show micropore-rich diameters <2 nm [34]. In general, the surface properties of kaolin in this study are typical and not substantially improved. Calcined kaolin had better surface properties than other samples. Even though in this study, acid-leached kaolin was impregnated, future investigations may consider using calcined kaolin as a support for catalyst impregnation.

### 3.3. TGA Analysis Results

**Figure 4:**
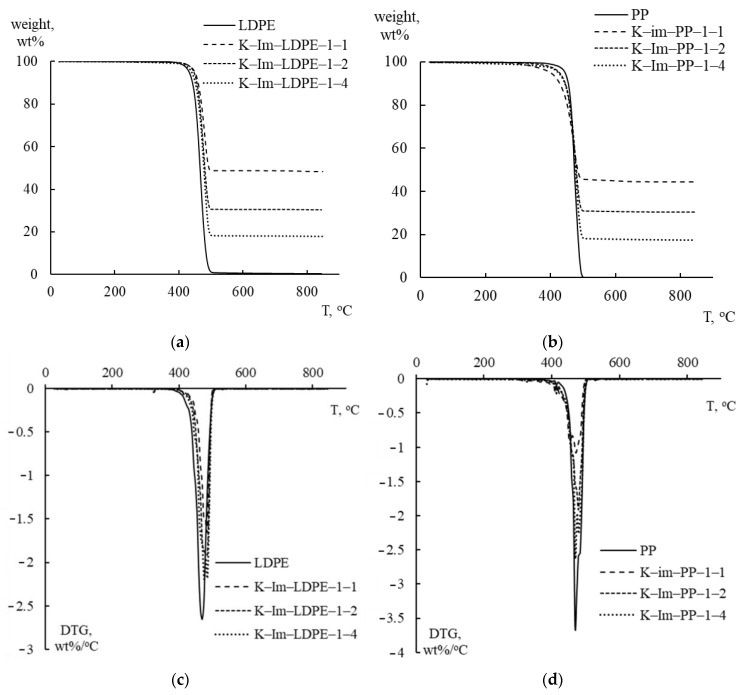
TGA and DTG analysis curves of LDPE and PP with acid-leached kaolin (K-Ac) and iron-impregnated kaolin (K-Im). (**a**) TGA: LDPE with K-Im, (**b**) TGA: PP with K-Im, (**c**) DTG: LDPE with K-Im, (**d**) DTG: PP with K-Im.

Figure 4 graphically summarizes the TGA and derivative thermogravimetry (DTG) analysis of PE and PP mixed with catalysts. PE and PP were mixed with iron-impregnated kaolin (K-Im) in proportions that varied from 1:4 (K-Im/LDPE 1:4 and K-Im/PP 1:4) to 1:2 (K-Im/LDPE 1:2 and K-Im/PP 1:2) and 1:1 (K-Im/LDPE 1:1 and K-Im/PP 1:1). As can be seen, the use of the modified kaolin did not substantially affect LDPE and PP decomposition. Both TGA and DTG follow the effect of the plastic-to-catalyst ratio proportionally. In both LDPE and PP, pyrolytic decomposition occurs in a single step between 420–430 °C and 490–500 °C. All tested samples had DTG curves with single peaks that occurred in the range of 470–485 °C, which is typical of LDPE and PP [35,36].

### 3.4. Product Distribution and Yield

**Figure 5:**
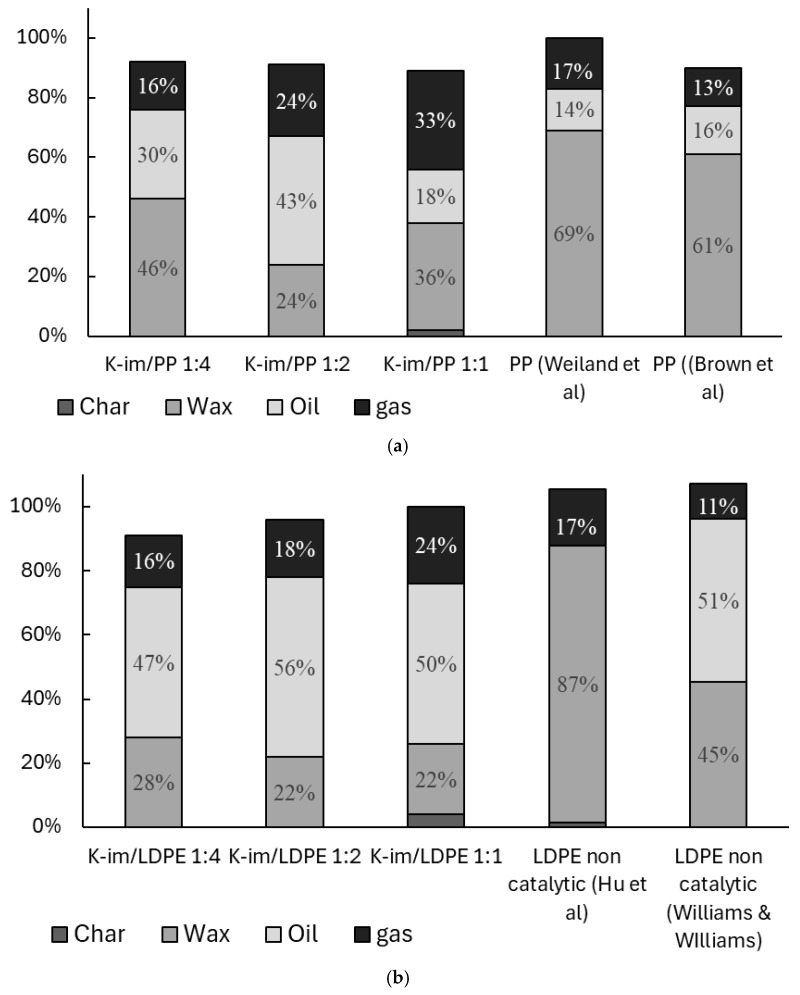
Balance of catalytic pyrolysis of (**a**) PP and (**b**) LDPE (wt. %) [37,38,41,42].

Figure 5 summarizes the yields of char, wax, oil, and gas from PP and LDPE catalytic pyrolysis and compares them with previous studies. Figure 5 is normalized after excluding the catalyst weight from the balance. The amounts indicated do not add up to 100% because of inevitable losses during sample collection that are not normalized. During the experiments, both PE and PP converted to gas, oil, and wax almost entirely. In general use, the K-Im catalyst allowed the oil yield and gas yield to increase compared to non-catalytic studies conducted by Weiland et al. and Brown et al. [37,38]. During the pyrolysis of PP, increasing catalyst loading led to a steady increase in gas yield, from 26 wt. % to 33 wt. % and 44 wt. % in K-Im/PP 1:4, K-Im/PP 1:2, and K-Im/PP 1:1 experiments, respectively. However, increasing the catalyst loading did not lead to a steady increase in oil yield. PP catalytic pyrolysis led to 30 wt. %, 43 wt. %, and 18 wt. % of oil yield in the K-Im/PP 1:4, K-Im/PP 1:2, and K-Im/PP 1:1 pyrolysis experiments, respectively. Wax yield first decreased with increasing catalyst load, from 46 wt. % to 24 wt. % in the K-Im/PP 1:4 and K-Im/PP 1:2 experiments, respectively. The increase in wax yield can be attributed to several possible mechanisms or their combination. First, there is the hydrothermal dealumination of the Si-Al matrix, when steam attacks framework Al–O–Si bonds above ~400 °C, ejecting Al and collapsing Brønsted acid sites. Experiments on zeolites demonstrated up to 80% loss of acidity after steaming [39]. Fewer strong acid sites lead to slowed-down β-scission and aromatization, allowing long C_20+_ chains survive to condense as wax. Second, reduced Fe enhances the water–gas–shift loop on its surface. Even trace steam lifts the H_2_/CO ratio in the micro-environment. Fischer–Tropsch studies with 4–10 mol. % H_2_O show lower CH_4_ and higher C_5_^+^ selectivity on Fe catalysts [40]. This increases chain-growth probability by stitching C_2_-C_3_ fragments back into C_20+_ wax. Third, H· atoms formed via H_2_O → ·OH + H· rapidly cap allylic radicals in the pyrolysis vapor. Early hydrogenation competes with secondary cracking. This keeps molecules long and saturated instead of letting them crack further into gasoline-range species. Complete Fe_2_O_3_ reduction only generates ~3 wt. % water—far too little to re-steam the whole vapor flow but enough to attack the acid sites and to sit right where reduced Fe performs FT-type coupling.

Figure 5b compares catalytic pyrolysis of LDPE with K-Im catalyst and previous non-catalytic experiment conducted by Hu et al. and Williams et al. [41,42]. Hu et al. demonstrated that most of the non-catalytic slow pyrolysis product is wax, reaching 87 wt. %. Wiliam et al. demonstrated that fast pyrolysis in a fluidized bed allows the cracking of roughly half of the wax, producing Py-oil and gas. K-Im/PP catalyst allowed a reduction in wax yield (to 22 wt. %) and a substantial increase in Py-oil (56 wt. %) and gas yields. In the case of LDPE catalytic pyrolysis increasing, the catalyst loading did not lead to notable changes in wax, char, or gas yield. Some of the char remained with the catalyst in the experiments with high catalyst loading, 2 wt. % and 4 wt. % in cases of K-Im/PP 1:1 and K-Im/LDPE 1:1, respectively. Gases that evolve from PP and LDPE pyrolysis contain H_2_, CO, CH4, and light alkanes and alkenes. These gases reduce Fe^3+^ to Fe^2+^/Fe^0^ and generate FeO, Fe, and Fe_3_C nanoparticles [43]. Further carbon formation mechanisms include catalytic cracking/dehydrogenation of olefins, Boudouard disproportionation ($2CO↔C+CO_{2})$, and Bosch-type CO hydrogenation ($CO+H_{2}\to C+H_{2}O$). Fe effectively dissociates C-O and C–H bonds and then extrudes C as graphite filaments. LDPE pyrolysis produces a gas mix richer in ethylene, and ethylene cracks to carbon on Fe far faster than propylene. The reduction of Fe_2_O_3_ causes the formation of oxygenated hydrocarbons and water in the product stream. Wax formation did not change substantially, suggesting its formation was limited to LDPE decomposition and not to the catalytic effect of Fe^3+^, Fe^2+^, or Fe^0^. This can be attributed to higher carbon deposition, confirmed by higher char yield in the mass balance that hinders catalytic activity. Ethylene is also much harder to couple at only 0.1–0.2 MPa and 500 °C, so the same Fe surface mostly dehydrogenates it to coke instead of building long liquids [44,45].

**Figure 6:**
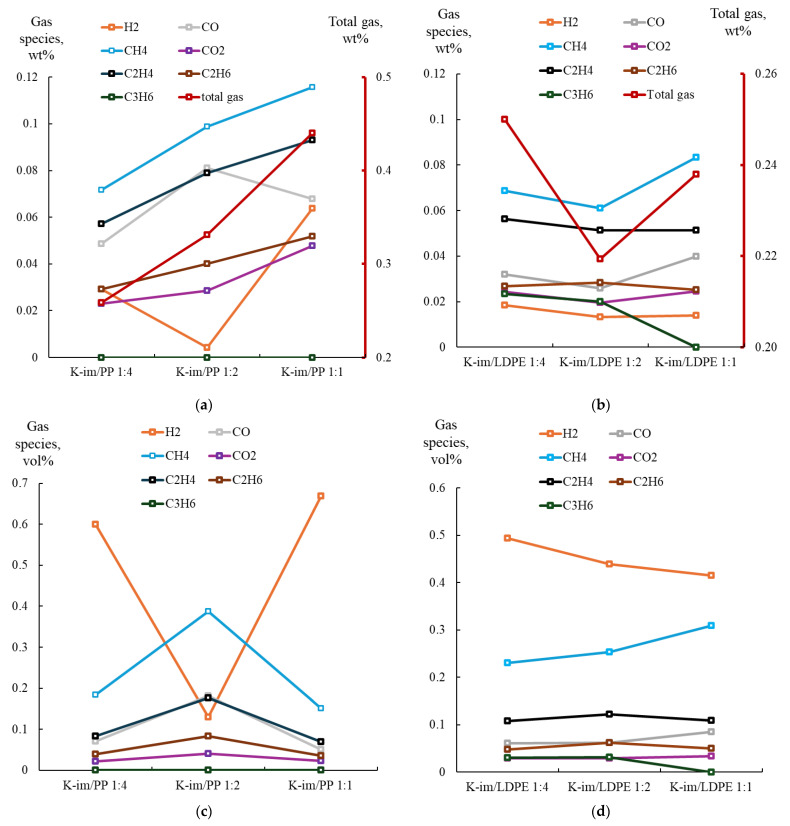
(**a**) Yield of PyGas and its components from K-Im/PP. (**b**) Yield of PyGas and its components from K-Im/LDPE. (**c**) Composition of PyGas from K-im/PP pyrolysis. (**d**) Composition of PyGas from K-im/LDPE pyrolysis.

Figure 6 summarizes the analysis of collected gas samples from PP (see Figure 6a for gases yield and Figure 6c for PyGas composition) and LDPE pyrolysis (see Figure 6b,d for PyGas composition). During PP pyrolysis the gas yield steadily increased with increasing K-Im/PP proportion, from 16 wt. % to 24 wt. % and 33 wt. %, in the K-Im/PP 1:4, K-Im/PP 1:2, and K-Im/PP 1:1 pyrolysis experiments, respectively. The main gas constituents were CH_4_, C_2_H_4_, and CO. The formation of oxygenated compounds, such as CO and CO_2_, consumed oxygen from the reduction of Fe_2_O_3_ to Fe_3_O_4_ and FeO [43]. The CO yield first increased due to increasing abundance of Fe_2_O_3_ in K-Im. However, increasing K-Im proportion apparently leads to the Boudouard disproportionation reaction simultaneously increasing char yield and CO_2_ yield. H_2_ yield first decreases from 0.029 to 0.004 wt. % when K-Im is increased from K-Im/PP 1:4 to K-Im/PP 1:2. This was also accompanied with increasing water content in the liquid products (see Tables S3–S5 in Supplementary Materials).

Figure 6b summarizes the yield of gases evolved from the catalytic pyrolysis process, illustrating gas yield when the K-Im catalyst-to-LDPE ratio was increased. During LDPE catalytic pyrolysis, increasing the ratio of K-Im catalyst led to a decrease in total gas yield from 0.25 wt. % to 0.22 wt. % but then led to an increase to 0.24 wt. % in K-Im/LDPE 1:4, K-Im/LDPE 1:2, and K-Im/LDPE 1:1, respectively. In general, the main components of the PyGas included CH_4_ and C_2_H_4_. This decrease was accompanied with an increase in water content in the oil, similarly to PP pyrolysis (see Tables S6–S8 in Supplementary Materials). Water content increased from 1.4% to 3.14% but then decreased to 0 in K-Im/LDPE 1:4, K-Im/LDPE 1:2, and K-Im/LDPE 1:1, respectively. Similarly to PP, Fe_2_O_3_ on the K-Im catalyst reduces to Fe_3_O_4_ and FeO, donating oxygen to hydrocarbon oxidation, producing CO, CO_2_, and H_2_O. CO and CO_2_ yield increases were accompanied with increases in char yield. However, while increasing the catalyst ratio in PP pyrolysis to K-Im/PP 1:1 led to a decrease in CO yield, in LDPE pyrolysis, increasing the K-Im/LDPE 1:1 led to an increase in CO yield.

### 3.5. Pyrolysis Oil Characteristics

**Figure 7:**
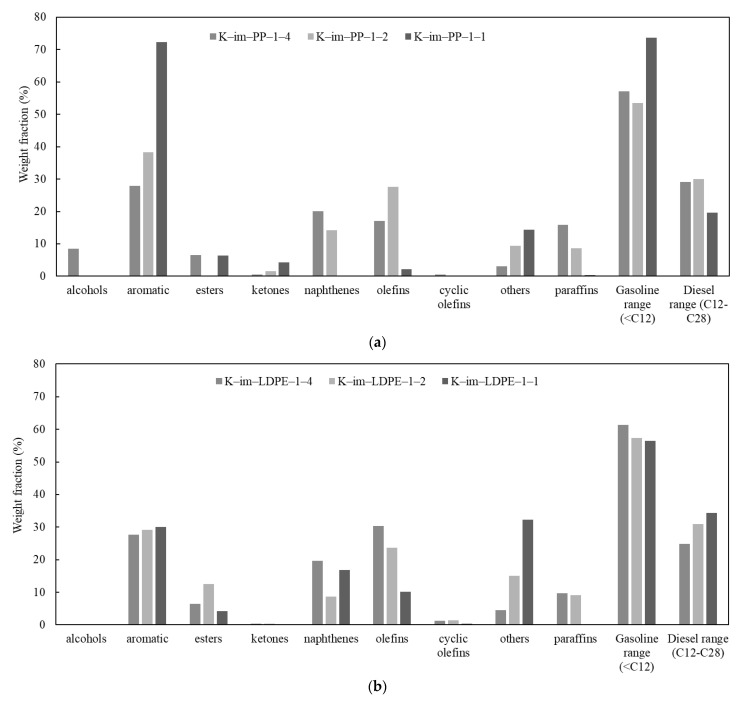
Summary of GC-MS analysis: (**a**) PP catalytic pyrolysis oil, (**b**) LDPE catalytic pyrolysis oil.

The detailed results of GC-MS analyses of Py-Oil are provided in Tables S3–S8 (see Supplementary Materials); their chromatograms are provided in Figure S3 (see Supplementary Materials). Figure 7a summarizes the relative percentage of Py-Oil derived from the K-Im/PP 1:4, K-Im/PP 1:2, and K-Im/PP 1:1 experiments. Primary PP cracking produces iso-olefins. A higher catalyst-to-PP ratio increases area/acid sites per gram vapor and vapor contact time; consequently, more secondary oligomerization–cyclisation–cracking cycles occur at Brønsted/Lewis sites, converting C_3_–C_6_ olefins into cyclic C_6_–C_10_ naphthenes. Fe_2_O_3_ reduction increases available Fe^0^ and Fe_3_C, which dehydrogenate naphthenes and olefins (example: $C_{6}H_{12}\to C_{6}H_{6}+3H_{2}$. Increasing the catalyst loading during catalytic pyrolysis of LDPE decreased the Py-oil yield but dramatically increased the aromatic content from 27.9% to 72.3%. At K-Im/PP 1:1, most of the aromatics were monoaromatic. Heterocyclics were nearly absent. The combined content of olefins and paraffins dropped from 32.9% to 2.5%, suggesting that steam and iron immediately reform the surviving paraffins. Because each new aromatic ring consumes six olefinic carbons but exports three H_2_, the liquid quickly skews toward low-H/C aromatics while paraffins and olefins collapse. Most of the produced oil components were in the gasoline range with carbon number < C12, which rose from 57.2% to 73.71% in the K-Im/PP 1:4 and K-Im/PP 1:1 experiments, respectively. Oil constituents also included oxygenated compounds such as ethers, ketones, and alcohols that formed as a result of iron oxide reduction. The LDPE Py-oil relative content is given in Figure 7b. Unlike PP pyrolysis, increasing the K-Im/LDPE ratio did not substantially increase the aromatic relative content. Paraffin, olefin, and napthene individual relative contents are reduced with a higher catalyst-to-LDPE ratio, and their combined relative content reduces from 61% to 33.3%. The category “others” includes mainly ethers whose content and carbon number increases with increasing K-Im/LDPE ratio. The range of the relative content of gasoline from LDPE catalytic pyrolysis was also high; however, it decreased with increasing K-Im/LDPE ratio. Simultaneously, the diesel range increased due to the increase in ethers with a high carbon number.

Catalyst deactivation due to coke deposition and iron oxide (Fe_2_O_3_) reduction are the two major hurdles during the catalytic pyrolysis of plastics with ferrous catalysts [31]. Fe(NO_3_)_3_-impregnated kaolin (≈6.6 wt. % Fe_2_O_3_ on a metakaolin support) is one example of a low-cost bifunctional catalyst used to enhance polyolefin pyrolysis, providing both acidic sites from the clay and redox functionality from iron. XRD analyses of iron-modified kaolin after PP and LDPE pyrolysis are presented in Table 4, where the samples are compared with iron-modified calcined kaolin. During the pyrolysis process, Fe_2_O_3_ (74.4 wt. %) was reduced to magnetite and ferrous oxide with non-stoichiometric proportions, Fe_2_._792_O_4_ and Fe_0_._942_O, respectively. The most straightforward regeneration method is high-temperature oxidation of coke deposits in an air (O_2_) atmosphere, which also facilitates the oxidation of iron, recovering redox sites. This method can achieve near-complete coke removal and full recovery of catalytic activity when properly controlled. For example, a coked zeolite catalyst (HZSM-5) used in plastic pyrolysis regained its initial activity and product selectivity after regeneration at 550 °C in O_2_ [8]. Fe-containing catalysts regenerated in air often show performance comparable to fresh samples [46]. Upon air regeneration, the Fe species re-oxidize (e.g., Fe^2+^/Fe^3^O_4_ formed during anaerobic pyrolysis revert to Fe^3+^/Fe_2_O_3_), restoring the catalyst’s original oxidation state and redox functionality. Since the catalyst was prepared from calcined kaolin converted to metakaolin, preservation of kaolin structural integrity is avoided. Nonetheless, repeated high-temperature cycles can induce some structural degradation over the long term. Luo et al. reused kaolin catalysts (10-micron size) four times and discovered that the yield of aromatic hydrocarbons increased with reuse due to an increase in acid sites that promote olefin aromatization. However, excessive coking will eventually block the active sites on the catalyst surface. Therefore, kaolin-derived catalysts can potentially be reused several times before regeneration is required. In the case of the poisoning of a catalyst with additives from plastics or loss of metal loading, acid washing and metal reimpregnation can be applied. Sintering of Fe oxide particles is a key concern—small Fe_2_O_3_ crystallites can agglomerate into larger clusters under thermal stress, reducing the active surface area [47]. For instance, Ni/Al_2_O_3_ catalysts showed significant surface area loss and activity decline after successive coke burn-offs due to metal particle growth and support damage. Fe/kaolin may similarly exhibit slightly diminished acidity or pore structure after many air–regen cycles, even if initial cycles restore performance. To mitigate thermal damage, coke combustion should be performed with controlled oxygen flow and moderate heating rates to avoid hot spots. Overall, thermal air oxidation is highly effective in coke removal (it often achieves >90–99% carbon removal and usually yields the highest recovery of catalyst activity among methods, making it the baseline technique in both laboratory and industrial settings (akin to the regenerator of an FCC unit) [46,48]. The trade-off is that each cycle contributes a bit of cumulative thermal stress; however, studies on Fe-based catalysts have shown stable performance over multiple air regenerations (e.g., <5% drop in olefin yield over five cycles with Fe/Mg-ZSM-5) [48].

The Fe(III)-impregnated kaolin used herein is intrinsically low-cost because (i) kaolin is a widely available commodity clay, previously highlighted as a low-cost, naturally abundant catalyst support for plastic pyrolysis [7,8,10]; (ii) the synthesis employs only standard unit operations (calcination, acid leaching, aqueous/alcoholic impregnation, drying) with commodity chemicals, avoiding templating agents, hydrothermal crystallization, or precious metals [31]; (iii) the catalyst is robust to high temperatures and can be regenerated by simple air calcination, extending the lifetime and reducing make-up rates. Notably, a modest iron loading (≈6.6 wt. % as Fe_2_O_3_) is sufficient to achieve the desired redox–acid synergy with kaolin’s Brønsted/Lewis sites, eliminating the need for surface-area engineering [31]; (iv) use of abundant Fe (also referred as base catalysts) instead of precious metals is explicitly mentioned as low-cost in previous review studies [10]. In combination with its demonstrated activity for both PP and LDPE in the same reactor, the material offers a favorable total cost of ownership relative to conventional zeolites or noble-metal catalysts.

## 4. Conclusions

Kaolin calcined, acid-leached, and impregnated with 6.6 wt. % Fe^3+^ delivers a low-cost catalyst that, despite its modest post-impregnation BET area of ≈10 m^2^ g^−1^, preserves the Si/Al framework required for Brønsted–Lewis acidity. During fixed-bed pyrolysis, the lattice oxygen of Fe_2_O_3_ and the structural OH of kaolin are stripped by hot polymer fragments; the catalyst therefore self-reduces to Fe^0^/Fe_3_C while ejecting H_2_O, CO, and H_2_, so PyGas concentrations in the effluent rise in direct proportion to the Fe/kaolin loading. This redox–acid synergy cuts the apparent first-order activation energy for polypropylene (PP) degradation to 154 kJ mol^−1^, compared with >300 kJ mol^−1^ for the uncatalyzed system, whereas LDPE kinetics remain well above 270 kJ mol^−1^. When the catalyst-to-PP ratio is stepped from 1:4 to 1:1, non-condensable gas climbs from 26 wt. % to 44 wt. %, mono-aromatics in the oil soar from 27.9% to 72.3%, and combined paraffins + olefins collapse to 2.5%, while wax shows a non-monotonic 46 → 24 → 38 wt. % profile as acid sites are first activated and later partially steamed out. The same catalyst acts quite differently on LDPE: at a 1:4 ratio, it suppresses wax to 22 wt. % and lifts oil to 56 wt. %, yet further loading mainly boosts CH_4_- and C_2_H_4_-rich PyGas and char without appreciably raising liquid aromatics. Thus, tuning Fe/kaolin dosage enables selective valorization—toward gasoline-range aromatic oil from PP or wax-reduced diesel-range oil plus methane-lean PyGas from LDPE—so the inexpensive, clay-based catalyst shows promise for scalable waste-plastic processing [8].

## Figures and Tables

**Table 1:** Elemental analyses of PP and LDPE compared to PET, PS, and PU (wt. %).

Name	C	H	N	S	O	Reference
PP	85.72 ± 0.05	14.22 ± 0.07	0	0.00	0.06 ± 0.06	This study
LDPE	85.65 ± 0.47	14.21 ± 0.03	0	0.00	0.14 ± 0.14	This study

**Table 2:** Summary of the iron loading onto the catalyst (wt. %).

	As-Received	Calcined	Acid-Leached	Impregnated
Kaolin	0.68	0.69	0.70	6.60

**Table 3:** Analyses of kaolin-derived material surface characteristics.

	As-Received			Calcined			Acid-Leached			Impregnated		
	SSA, m^2^·g^−1^	Pore V, cm^3^·g^−1^	Pore S, nm	SSA, m^2^·g^−1^	Pore V, cm^3^·g^−1^	Pore S, nm	SSA, m^2^·g^−1^	Pore V, cm^3^·g^−1^	Pore S, nm	SSA, m^2^·g^−1^	Pore V, cm^3^·g^−1^	Pore S, nm
Kaolin	11.09	0.038	1.59	57.36	0.14	2.72	11.7	0.026	0.7	10.41	0.034	0.7

**Table 4:** XRD analysis results of iron-modified calcined kaolin catalyst before pyrolysis and after LDPE and PP pyrolysis experiments.

	Analyte	K, Fe-Impregnated, wt. %	K, Impregnated, After PP Pyrolysis, wt. %	K, Impregnated, After LDPE Pyrolysis, wt. %
Alumina	ɣ-Al_2_O_3_	9.6	13.5%	30.3%
Quartz	SiO_2_	12.0	27.5%	24.5%
Hematite	Fe_2_O_3_	74.4		
Iron oxide	Fe_2_._792_O_4_		33.0%	41.3%
Iron oxide	Fe_0_._942_O		11.1%	
Anatase	TiO	4.0	4.9%	3.9%

## Data Availability

The original contributions presented in this study are included in the article/Supplementary Material. Further inquiries can be directed to the corresponding author.

## Supplementary Materials

The following supporting information can be downloaded at https://www.mdpi.com/article/10.3390/polym17212963/s1. Figure S1. Catalytic material preparation procedure; Figure S2. XRD analyses of catalytic materials: (a) kaolin, as received, wt. %; (b) kaolin, calcined; (c) kaolin, acid-leached; (d) kaolin, impregnated; Figure S3. (a) GC-MS chromatogram of oil produced during catalytic pyrolysis of Fe(III) modified kaolin with PP with rate 1:1 (b) GC-MS chromatogram of oil produced during catalytic pyrolysis of Fe(III) modified kaolin with PP with rate 1:2 (c) GC-MS chromatogram of oil produced during catalytic pyrolysis of Fe(III) modified kaolin with PP with rate 1:4 (d) GC-MS chromatogram of oil produced during catalytic pyrolysis of Fe(III) modified kaolin with LDPE with rate 1:1 (e) GC-MS chromatogram of oil produced during catalytic pyrolysis of Fe(III) modified kaolin with LDPE with rate 1:2 (f) GC-MS chromatogram of oil produced during catalytic pyrolysis of Fe(III) modified kaolin with LDPE with rate 1:4; Table S1. XRF analyses results of kaolin as received, kaolin calcined, acid-leached kaolin, Fe-impregnated kaolin; Table S2. XRD analyses of kaolin as received, kaolin calcined, acid-leached kaolin, and Fe-impregnated kaolin catalyst after PP pyrolysis and after LDPE pyrolysis; Table S3. GC-MS analysis result of oil produced during catalytic pyrolysis of Fe(III)-modified kaolin with PP with a rate 1:1; Table S4. GC-MS analysis result of oil produced during catalytic pyrolysis of Fe(III)-modified kaolin with PP with rate 1:2; Table S5. GC-MS analysis results for oil produced during catalytic pyrolysis of Fe(III)-modified kaolin with PP with rate 1:4; Table S6. GC-MS analysis results for oil produced during catalytic pyrolysis of Fe(III)-modified kaolin with LDPE with rate 1:1; Table S7. GC-MS analysis result of oil produced during catalytic pyrolysis of Fe(III)-modified kaolin with LDPE with rate 1:2; Table S8. GC-MS analysis result of oil produced during catalytic pyrolysis of Fe(III)-modified kaolin with LDPE with rate 1:1.

## Abbreviations

The following abbreviations are used in this manuscript:

PyGas	Pyrolysis gas
PP	Polypropylene
PE	Polyethylene
LDPE	Low-density polyethylene
PS	Polystyrene
PET	Polyethylene terephthalate
PUR	Polyurethane
ZSM-5	Zeolite Socony Mobil–5
BET	Brunauer–Emmett–Teller
XRD	X-ray diffraction
XRF	X-ray fluorescence
GC-MS	Gas chromatography–mass spectrometry
GC-TCD	Gas chromatography thermal conductivity detector
DFT	Density functional theory
TGA	Thermogravimetric analysis
DTG	Differential thermogravimetric analysis
K-Im	Kaolin-impregnated
PILCs	Pillared clays

## References

[1] Laghezza M., Fiore S., Berruti F. (2024). A Review on the Pyrolytic Conversion of Plastic Waste into Fuels and Chemicals. J. Anal. Appl. Pyrolysis.

[2] Charitopoulou M.-A., Koutroumpi S., Achilias D.S. (2025). Thermal Characterization and Recycling of Polymers from Plastic Packaging Waste. Polymers.

[3] Dimitrov N., Kratofil Krehula L., Ptiček Siročić A., Hrnjak-Murgić Z. (2013). Analysis of Recycled PET Bottles Products by Pyrolysis-Gas Chromatography. Polym. Degrad. Stab..

[4] Inayat A., Fasolini A., Basile F., Fridrichova D., Lestinsky P. (2022). Chemical Recycling of Waste Polystyrene by Thermo-Catalytic Pyrolysis: A Description for Different Feedstocks, Catalysts and Operation Modes. Polym. Degrad. Stab..

[5] Eschenbacher A., Varghese R.J., Weng J., Van Geem K.M. (2021). Fast Pyrolysis of Polyurethanes and Polyisocyanurate with and without Flame Retardant: Compounds of Interest for Chemical Recycling. J. Anal. Appl. Pyrolysis.

[6] Nurlybayeva A., Yermekova A., Taubayeva R., Sarova N., Sapiyeva A., Mateeva S., Matniyazova G., Bulekbayeva K., Jetpisbayeva G., Tamabekova M. (2025). Modern Methods of Obtaining Synthetic Oil from Unconventional Hydrocarbon Raw Materials: Technologies, Catalysts, and Development Prospects. Polymers.

[7] Seliverstov E.S., Furda L.V., Lebedeva O.E. (2022). Thermocatalytic Conversion of Plastics into Liquid Fuels over Clays. Polymers.

[8] Eldahshory A.I., Emara K., Abd-Elhady M.S., Ismail M.A. (2023). Catalytic Pyrolysis of Waste Polypropylene Using Low-Cost Natural Catalysts. Sci. Rep..

[9] Kumar R., Sadhukhan A.K., Singh R.K., Ruj B., Gupta P. (2024). Investigations on the Effect of Kaolin Catalyst on the Yield of Various Products Obtained from Pyrolysis of Low-Density Polyethylene (LDPE) Wastes and Reaction Kinetics. Environ. Sci. Pollut. Res..

[10] Peng Y., Wang Y., Ke L., Dai L., Wu Q., Cobb K., Zeng Y., Zou R., Liu Y., Ruan R. (2022). A Review on Catalytic Pyrolysis of Plastic Wastes to High-Value Products. Energy Convers. Manag..

[11] Luo W., Hu Q., Fan Z., Wan J., He Q., Huang S., Zhou N., Song M., Zhang J., Zhou Z. (2020). The Effect of Different Particle Sizes and HCl-Modified Kaolin on Catalytic Pyrolysis Characteristics of Reworked Polypropylene Plastics. Energy.

[12] Panda A.K., Singh R.K. (2013). Experimental Optimization of Process for the Thermo-Catalytic Degradation of Waste Polypropylene to Liquid Fuel. Adv. Energy Eng..

[13] Luo W., Fan Z., Wan J., Hu Q., Dong H., Zhang X., Zhou Z. (2021). Study on the Reusability of Kaolin as Catalysts for Catalytic Pyrolysis of Low-Density Polyethylene. Fuel.

[14] Kumar R., Sadhukhan A.K., Gupta P., Singh R.K., Ruj B. (2024). Recovery of Enhanced Gasoline-Range Fuel from Catalytic Pyrolysis of Waste Polypropylene: Effect of Heating Rate, Temperature, and Catalyst on Reaction Kinetics, Products Yield, and Compositions. Process Saf. Environ. Prot..

[15] Rahman M., Faruk M.O., Islam M.W., Akter M., Saha J.K., Ahmed N., Sharmin A., Hoque M.d.A., Afroze M., Khan M. (2024). Comparison of the Effect of Kaolin and Bentonite Clay (Raw, Acid-Treated, and Metal-Impregnated) on the Pyrolysis of Waste Tire. ACS Omega.

[16] Budsaereechai S., Hunt A.J., Ngernyen Y. (2019). Catalytic Pyrolysis of Plastic Waste for the Production of Liquid Fuels for Engines. RSC Adv..

[17] Tasleem S., Soliman A., Alsharaeh E.H. (2025). Recent Developments in Catalytic Materials and Reactors for the Catalytic Pyrolysis of Plastic Waste into Hydrogen: A Critical Review with a Focus on the Circular Economy. RSC Adv..

[18] Lee W.-T., Bobbink F.D., Van Muyden A.P., Lin K.-H., Corminboeuf C., Zamani R.R., Dyson P.J. (2021). Catalytic Hydrocracking of Synthetic Polymers into Grid-Compatible Gas Streams. Cell Rep. Phys. Sci..

[19] Kohli K., Chandrasekaran S.R., Prajapati R., Kunwar B., Al-Salem S., Moser B.R., Sharma B.K. (2022). Pyrolytic Depolymerization Mechanisms for Post-Consumer Plastic Wastes. Energies.

[20] Boukhemkhem A., Rida K., Pizarro A.H., Molina C.B., Rodriguez J.J. (2019). Iron Catalyst Supported on Modified Kaolin for Catalytic Wet Peroxide Oxidation. Clay Miner..

[21] Li K., Lei J., Yuan G., Weerachanchai P., Wang J.-Y., Zhao J., Yang Y. (2017). Fe-, Ti-, Zr- and Al-Pillared Clays for Efficient Catalytic Pyrolysis of Mixed Plastics. Chem. Eng. J..

[22] Akin O., Varghese R.J., Eschenbacher A., Oenema J., Abbas-Abadi M.S., Stefanidis G.D., Van Geem K.M. (2023). Chemical Recycling of Plastic Waste to Monomers: Effect of Catalyst Contact Time, Acidity and Pore Size on Olefin Recovery in Ex-Situ Catalytic Pyrolysis of Polyolefin Waste. J. Anal. Appl. Pyrolysis.

[23] Fekhar B., Zsinka V., Miskolczi N. (2019). Value Added Hydrocarbons Obtained by Pyrolysis of Contaminated Waste Plastics in Horizontal Tubular Reactor: In Situ Upgrading of the Products by Chlorine Capture. J. Clean. Prod..

[24] Singh R.K., Ruj B., Sadhukhan A.K., Gupta P. (2022). Conventional Pyrolysis of Plastic Waste for Product Recovery and Utilization of Pyrolytic Gases for Carbon Nanotubes Production. Environ. Sci. Pollut. Res..

[25] Wu L., Ma H., Mei J., Li Y., Xu Q., Li Z. (2022). Low Energy Consumption and High Quality Bio-Fuels Production via in-Situ Fast Pyrolysis of Reed Straw by Adding Metallic Particles in an Induction Heating Reactor. Int. J. Hydrogen Energy.

[26] Fekhar B., Gombor L., Miskolczi N. (2019). Pyrolysis of Chlorine Contaminated Municipal Plastic Waste: In-Situ Upgrading of Pyrolysis Oils by Ni/ZSM-5, Ni/SAPO-11, Red Mud and Ca(OH)_2_ Containing Catalysts. J. Energy Inst..

[27] Ma Y., Gao N., Quan C., Sun A., Olazar M. (2024). High-Yield H_2_ Production from HDPE through Integrated Pyrolysis and Plasma-Catalysis Reforming Process. Chem. Eng. J..

[28] Aminu I., Nahil M.A., Williams P.T. (2022). Hydrogen Production by Pyrolysis–Nonthermal Plasma/Catalytic Reforming of Waste Plastic over Different Catalyst Support Materials. Energy Fuels.

[29] Wang W., Ma Y., Chen G., Quan C., Yanik J., Gao N., Tu X. (2022). Enhanced Hydrogen Production Using a Tandem Biomass Pyrolysis and Plasma Reforming Process. Fuel Process. Technol..

[30] Xu Z., Gao N., Ma Y., Wang W., Quan C., Tu X., Miskolczi N. (2023). Biomass Volatiles Reforming by Integrated Pyrolysis and Plasma-Catalysis System for H_2_ Production: Understanding Roles of Temperature and Catalyst. Energy Convers. Manag..

[31] Cai N., Li X., Xia S., Sun L., Hu J., Bartocci P., Fantozzi F., Williams P.T., Yang H., Chen H. (2021). Pyrolysis-Catalysis of Different Waste Plastics over Fe/Al_2_O_3_ Catalyst: High-Value Hydrogen, Liquid Fuels, Carbon Nanotubes and Possible Reaction Mechanisms. Energy Convers. Manag..

[32] Rouibah K., Ferkous H., Abdessalam-Hassan M., Mossab B.L., Boublia A., Pierlot C., Abdennouri A., Avramova I., Alam M., Benguerba Y. (2024). Exploring the Efficiency of Algerian Kaolinite Clay in the Adsorption of Cr(III) from Aqueous Solutions: Experimental and Computational Insights. Molecules.

[33] Kwon S., Hwang H., Lee Y. (2019). Effect of Pressure Treatment on the Specific Surface Area in Kaolin Group Minerals. Crystals.

[34] David M.K., Okoro U.C., Akpomie K.G., Okey C., Oluwasola H.O. (2020). Thermal and Hydrothermal Alkaline Modification of Kaolin for the Adsorptive Removal of Lead(II) Ions from Aqueous Solution. SN Appl. Sci..

[35] Meena P., Bhoi R. (2025). Thermodynamic and Kinetic Analysis of Waste Plastic Pyrolysis: Synergistic Effects and Sustainability Perspectives. Next Sustain..

[36] Dubdub I., Al-Yaari M. (2020). Pyrolysis of Low Density Polyethylene: Kinetic Study Using TGA Data and ANN Prediction. Polymers.

[37] Weiland F., Qureshi M.S., Wennebro J., Lindfors C., Ohra-aho T., Shafaghat H., Johansson A.-C. (2021). Entrained Flow Gasification of Polypropylene Pyrolysis Oil. Molecules.

[38] Brown J.L., Brown R.C., Cecon V.S., Vorst K., Smith R.G., Daugaard T.J. (2024). Increasing Pyrolysis Oil Yields and Decreasing Energy Consumption via Thermal Oxo-Degradation of Polyolefins. Cell Rep. Phys. Sci..

[39] Jin L., Hu H., Zhu S., Ma B. (2010). An Improved Dealumination Method for Adjusting Acidity of HZSM-5. Catal. Today.

[40] Wang Z., Mai K., Kumar N., Elder T., Groom L.H., Spivey J.J. (2017). Effect of Steam During Fischer–Tropsch Synthesis Using Biomass-Derived Syngas. Catal. Lett..

[41] Hu W., Sárossy Z., Jensen A.D., Daugaard A.E., Jensen P.A. (2023). Two-Stage Fixed-Bed Low-Density Polyethylene Pyrolysis: Influence of Using Different Catalytic Materials in the Second Stage. Energy Fuels.

[42] Williams P.T., Williams E.A. (1999). Fluidised Bed Pyrolysis of Low Density Polyethylene to Produce Petrochemical Feedstock. J. Anal. Appl. Pyrolysis.

[43] Lin Z., Liu J., Li L., Cai H., Lin S., Evrendilek F., Chen S., Chen X., Chen T., He Y. (2024). Fe_2_O_3_, Al_2_O_3_, or Sludge Ash-Catalyzed Pyrolysis of Typical 3D Printing Waste toward Tackling Plastic Pollution. J. Hazard. Mater..

[44] Heveling J., Nicolaides C.P., Scurrell M.S. (1998). Catalysts and Conditions for the Highly efficient, Selective and Stable Heterogeneous Oligomerisation of Ethylene. Appl. Catal. A Gen..

[45] Wang M. (2020). Research Progress of Iron-Based Catalysts for Selective Oligomerization of Ethylene. RSC Adv..

[46] Li Y., Liu T., Deng S., Liu X., Meng Q., Tang M., Wu X., Zhang H. (2024). Surface Modification of Fe-ZSM-5 Using Mg for a Reduced Catalytic Pyrolysis Temperature of Low-Density Polyethylene to Produce Light Olefin. Catalysts.

[47] Daligaux V., Richard R., Manero M.-H. (2021). Deactivation and Regeneration of Zeolite Catalysts Used in Pyrolysis of Plastic Wastes—A Process and Analytical Review. Catalysts.

[48] López A., De Marco I., Caballero B.M., Adrados A., Laresgoiti M.F. (2011). Deactivation and Regeneration of ZSM-5 Zeolite in Catalytic Pyrolysis of Plastic Wastes. Waste Manag..

