# Supplementary material for: Polyethylene and Polypropylene Pyrolysis Using Fe3+-Modified Kaolin Catalyst for Enhanced Gas and Pyrolysis Oil Production
Source: Polymers (Basel). 2025 Nov 6;17(21):2963. doi: 10.3390/polym17212963 (PMC12609207; doi:10.3390/polym17212963)
Supplement: Supplementary file 1 [file polymers-17-02963-s001.zip › polymers-3884638-supplementary.pdf]

polymers

Supplementary Materials: Polyethylene and Polypropylene Pyrolysis Using Fe^{3+}-Modified Kaolin Catalyst for Enhanced Gas and Pyrolysis Oil Production

Sergey Nechipurenko [1], Binara Dossumova [1], Sergey Efremov [1], Nazar Zabara [1], Aigerim Kaiaidarova [1], Olga Ibragimova [1], Anara Omarova [1], Fedor Pogorov [1] and Diyar Tokmurzin [2,*]

1 Center of Physical Chemical Methods of Research and Analysis, Faculty of Chemistry and Chemical Technology, Al-Farabi Kazakh National University, Almaty 050012, Kazakhstan
2 Laboratory for Chemical Technology, Ghent University, Industriele Scheikunde, Technologiepark-Zwijnaarde 125, 9052 Gent, Belgium
* Correspondence: tokmurzindiyar@gmail.com; Tel.: +32-471-692-478

Table S1. XRF analyses results of kaolin as received, kaolin-calcined, kaolin acid leached, kaolin Fe impregnated.

Analyte	K-as received	K-calcined	K-acid leached	K-Fe-impregnated
O	60.811	52.626	50.148	48.901
Na	0.028			0.034
Mg	0.022	0.047	0.048	0.03
Al	17.25	18.655	17.665	16.608
Si	20.213	21.452	20.651	19.441
P	0.045	0.047	0.012	0.013
S	0.005	0.003	0.003	0.005
Cl			0.027	0.012
K	0.075	0.076	0.092	0.07
Ca		0.041	0.066	0.079
Ti	0.842	0.844	0.901	0.702
Cr				0.019
Sc			0.017	
V	0.007	0.014		
Fe	0.683	0.693	0.697	6.602
Ni				0.017
Zn	0.005			0.004
Cu			0.007	
Ga	0.003	0.003	0.003	0.003
Sr	0.004	0.003		
Zr	0.002	0.002	0.002	
Pb	0.005	0.006	0.005	0.006

Table S2. XRD analyses of kaolin as received, kaolin-calcined, kaolin acid leached, kaolin Fe impregnated, kaolin impregnated after PP pyrolysis, and kaolin.

	Analyte	K, as received, wt%	K, calcined, wt%	K, acid leached, wt%	K, impregnated, wt%	K, impregnated after PP pyrolysis	K, impregnat ed after LDPE pyrolysis
Kaolinite	$Al_2Si_2O_5(OH)_4$	97.5					
Alumina	γ-Al_2O_3		26.9	25	9.6	13.5%	30.3%
Pyrophyllite	$Al_2(Si_4O_{10})O$		25.3	21.4			
Silica	SiO_2		21.3	16.1			
Hercynite	$Fe(Al_2O_4)$		13.4	9			
Quartz	SiO_2	2.5	13.1	28.5	12.0	27.5%	24.5%

Hercynite	$Fe(Al_2O_4)$			9.0			
Hematite	Fe_2O_3				74.4		
Iron oxide	$Fe_{2.792}O_4$					33.0%	41.3%
Iron oxide	$Fe_{0.942}O$					11.1%	
Anatase	TiO				4.0	4.9%	3.9%

Table S3. GC-MS analysis result of oil produced during catalytic pyrolysis of Fe(III) modified kaolin with PP with rate 1:1.

RT	Name	Match, %	Area	ω, %	Formula	Category	Group	Oxygen content
4.09	Propene	92.7	18089.1	0.13	C3H6	Alkene	olefins	
4.67	Water	92.5	267236.2	1.94	H2O	Inorganic solvent	others	0.017229
4.93	3,4-Dimethyldihydrofuran-2,5-dione	88.4	42754.4	0.31	C6H10O3	Cyclic anhydride	others	0.0011433
4.99	1,2,4,5-Tetroxane, 3,3,6,6-tetramethyl-	90.8	20653.5	0.15	C8H16O4	Organic peroxide	others	0.0005448
5.09	Cyclobutene	87.3	25518.5	0.19	C4H6	Cycloalkene	cyclic olefins	0
5.25	Acetaldehyde	93.7	328149.5	2.38	C2H4O	Aldehyde	others	0.0086436
6.83	1-Pentene	88.8	61341.5	0.45	C5H10	Alkene	olefins	0
6.97	Propanal, 2-methyl-	86.6	48113.2	0.35	C4H8O	Aldehyde	others	0.0007766
12.54	Benzene	99.4	1834852.4	13.33	C6H6	Aromatic hydrocarbon	aromatic	0
12.77	Cyclobutanone, 2,3,3-trimethyl-	87.2	34111.2	0.25	C7H12O	Cyclic ketone	ketones	0.0003566
12.91	3-Pentanone, 2-methyl-	82	29444.4	0.21	C6H12O	Ketone	ketones	0.0003354
15.43	Toluene	98.9	591238.4	4.29	C7H8	Alkylâ€‘benzene	aromatic	0
16.38	Propane, 2,2'-[ethylidenebis(oxy)]bis-	88.1	64848.7	0.47	C8H18O2	Diether / acetal	others	0.0010285
17.68	Ethylbenzene	98.3	380571.7	2.76	C8H10	Alkylâ€‘benzene	aromatic	0
17.81	Pentane, 2,2-dimethyl-	81.7	12540.4	0.09	C7H16	Branched alkane	parafins	0
18.47	Styrene	99.5	4046594.2	29.39	C8H8	Vinylâ€‘aromatic	aromatic	0
19.73	1-Hepten-3-one	82.5	41917.7	0.3	C7H12O	Unsaturated ketone	ketones	0.0004279
20.23	.alpha.-Methylstyrene	96.5	398913.3	2.9	C9H10	Vinylâ€‘aromatic	aromatic	0
20.49	Benzaldehyde	97	196943.3	1.43	C7H6O	Aromatic aldehyde	aromatic	0.0021558
21.19	Indane	82.3	35150.3	0.26	C9H10	Polycyclic aromatic hydrocarbon	aromatic	0
21.45	2-Vinylfuran	80.3	32986.6	0.24	C6H6O	Heteroâ€‘aromatic	aromatic	0.000408
21.56	1-Heptene, 5-methyl-	84.7	56689.1	0.41	C8H16	Branched alkene	olefins	0
21.63	Hexane, 3,3-dimethyl-	80.1	31964.4	0.23	C8H18	Branched alkane	parafins	0
21.83	Indene	94.4	116931.3	0.85	C9H8	Polycyclic aromatic hydrocarbon	aromatic	0
22.49	Acetophenone	95.9	333528.6	2.42	C8H8O	Aromatic ketone	ketones	0.0032224
23.23	1-Octene, 6-methyl-	80.1	86180.2	0.63	C9H18	Branched alkene	olefins	0
24.01	Naphthalene, 1,2-dihydro-	85.3	20550.5	0.15	C10H10	Partially hydrogenated PAH	aromatic	0

24.56	Naphthalene	99.3	1034696.1	7.52	C10H8	Polycyclic aromatic hydrocarbon	aromatic	0
24.78	1-Octene, 6-methyl-	83.7	73321.3	0.53	C9H18	Branched alkene	olefins	0
25.72	Carbonic acid, eicosyl vinyl ester	83.8	482071.7	3.5	C23H44O3	Carbonate ester (long chain)	esters	0.0045575
26.69	Naphthalene, 2-methyl-	85.2	75715	0.55	C11H10	Alkylâ€‘PAH	aromatic	0
27.62	Biphenyl	97.8	513229.3	3.73	C12H10	Aromatic hydrocarbon	aromatic	0
28.48	Diphenylmethane	86.1	107518.9	0.78	C13H12	Aromatic hydrocarbon	aromatic	0
29.78	Acenaphthylene	91.9	145209.2	1.05	C12H8	Polycyclic aromatic hydrocarbon	aromatic	0
29.88	Oxalic acid, allyl octadecyl ester	84.3	404384.5	2.94	C23H42O4	Ester (oxalate)	esters	0.0049178
32.96	Fluorene	82.3	60894.4	0.44	C13H10	Polycyclic aromatic hydrocarbon	aromatic	0
33.78	Benzene, 1,1'-(1,3-propanediyl)bis-	95	299327.7	2.17	C15H16	Diaryl alkane	aromatic	0
34.15	Benzophenone	94.2	156014.1	1.13	C13H10O	Aromatic ketone	ketones	0.0009921
35.2	Decyl octyl ether	88	1124870.8	8.17	C18H38O	Dialkyl ether	others	0.0048322
36.29	(E)-Stilbene	81.5	58970	0.43	C14H12	Diarylethene	aromatic	0
39.02	5H-Tetrazol-5-amine	86.4	73738.1	0.54	C2H3N5	Nâ€‘heteroaromatic	others	0
			13767774					0.0515715

Table S4. GC-MS analysis result of oil produced during catalytic pyrolysis of Fe(III) modified kaolin with PP with rate 1:2.

RT	Name	Match, %	Area	ω, %	Formula	Category	Group	Oxygen content
4.67	Water	92.5	496106.4	2.34	H2O	Inorganic solvent	others	0.020774
4.93	1-Butene	95.6	275809.8	1.30	C4H8	Alkene	olefins	0
4.98	1,2,4,5-Tetroxane, 3,3,6,6-tetramethyl-	84.8	79047.4	0.37	C8H16O4	Organic peroxide	others	0.001354
5.09	1,3-Butadiene	96.6	150900.3	0.71	C4H6	Conjugated diene	olefins	0
5.2	2-Butene, (E)-	94.5	77249.6	0.36	C4H8	Alkene	olefins	0
5.45	1-Propene, 2-methyl-	90.8	71406.9	0.34	C4H8	Alkene	olefins	0
6.82	1-Pentene	93.9	357993	1.69	C5H10	Alkene	olefins	0
6.97	Pentane	95.1	195276.5	0.92	C5H12	Alkane	parafins	0
7.28	Oxetane, 3-(1-methylethyl)-	90.9	80486.5	0.38	C6H12O	Cyclic ether	others	0.000606
10.19	2-Hexene, (E)-	88.3	103493.2	0.49	C6H12	Alkene	olefins	0
12.54	Benzene	99.2	1569494	7.40	C6H6	Aromatic hydrocarbon	aromatic	0
12.73	7-Azabicyclo[4,2,0]octan-8-one	83.7	46434.2	0.22	C8H11NO	Bicyclic ketone (lactam)	ketones	0.000255
12.77	1-Heptene	93.7	257908.2	1.22	C7H14	Alkene	olefins	0
12.91	Heptane	96	163946.5	0.77	C7H16	Alkane	parafins	0
13.87	Furan, 2-methoxy-	82.3	16403	0.08	C5H7O2	Heteroâ€‘aromatic ether	aromatic	0.00025

15.43	Toluene	97.9	611190.2	2.88	C7H8	Aromatic hydrocarbon	aromatic	0
15.54	Octane	95.8	115533.6	0.54	C8H18	Alkane	parafins	0
17.68	Ethylbenzene	94.4	288216.4	1.36	C8H10	Aromatic hydrocarbon	aromatic	0
17.71	1-Nonene	95	201466.5	0.95	C9H18	Alkene	olefins	0
17.81	Nonane	95.3	144403.5	0.68	C9H20	Alkane	parafins	0
17.85	Benzene, 1,3-dimethyl-	88.6	65730.6	0.31	C8H10	Aromatic hydrocarbon	aromatic	0
18.47	Styrene	97.7	1353673	6.38	C8H8	Vinylâ€'aromatic	aromatic	0
19.73	1-Decene	96.7	369719.5	1.74	C10H20	Alkene	olefins	0
19.81	Decane	95.1	250135.7	1.18	C10H22	Alkane	parafins	0
19.97	2-Cyclopropen-1-one, 2,3-diphenyl-	83.1	275575.3	1.30	C15H12O	Aromatic ketone	ketones	0.000998
20.23	.alpha.-Methylstyrene	92.4	149722	0.71	C9H10	Vinylâ€'aromatic	aromatic	0
20.56	Pimelic acid, di(3-phenylpropyl) ester	85.1	41134.5	0.19	C23H28O4	Diester	esters	0.000337
21.18	Benzeneethanol, .beta.-ethenyl-	83.7	35623.8	0.17	C9H10O	Aromatic alcohol	alcohols	0.0002
21.56	Cyclopropane, octyl-	97.7	422948.8	1.99	C11H22	Cycloalkane	naphthenes	0
21.63	Undecane	97.3	299069.5	1.41	C11H24	Alkane	parafins	0
21.83	Indene	96.1	220542	1.04	C9H8	Polycyclic aromatic hydrocarbon	aromatic	0
23.23	1-Dodecene	97.6	426614	2.01	C12H24	Alkene	olefins	0
23.29	Dodecane	93.1	352372.4	1.66	C12H26	Alkane	parafins	0
23.65	2-Methylindene	90.3	84120.2	0.40	C10H10	Polycyclic aromatic hydrocarbon	aromatic	0
23.81	1H-Indene, 3-methyl-	89.1	78975	0.37	C10H10	Polycyclic aromatic hydrocarbon	aromatic	0
24.01	Naphthalene, 1,2-dihydro-	84.6	49110.2	0.23	C10H12	Partially hydrogenated PAH	aromatic	0
24.14	1H-Indene, 1-(phenylmethylene)-	82.4	82051.5	0.39	C16H12	Polycyclic aromatic hydrocarbon	aromatic	0
24.57	Naphthalene	99.5	2074369	9.78	C10H8	Polycyclic aromatic hydrocarbon	aromatic	0
24.78	1-Tridecene	93.3	842185.8	3.97	C13H26	Alkene	olefins	0
24.83	Decane, 2,9-dimethyl-	87.7	326943.1	1.54	C12H26	Branched alkane	parafins	0
25.96	Naphthalene, 2-methyl-	84.3	25264.4	0.12	C11H10	Alkylâ€'PAH	aromatic	0
26.3	Cyclotetradecane	92.4	805129.8	3.80	C14H28	Cycloalkane	naphthenes	0
26.35	Naphthalene, 2-methyl-	89.7	175373.3	0.83	C11H10	Alkylâ€'PAH	aromatic	0
26.69	1H-Indene, 1-ethylidene-	89.2	154512.3	0.73	C11H10	Polycyclic aromatic hydrocarbon	aromatic	0
27.62	Biphenyl	96.8	497783.6	2.35	C12H10	Aromatic hydrocarbon	aromatic	0
27.95	Cyclododecane	88.9	1054636	4.97	C12H24	Cycloalkane	naphthenes	0
28.88	Naphthalene, 2-ethenyl-	88.5	81075.3	0.38	C12H10	Vinylâ€'PAH	aromatic	0
29.78	Acenaphthylene	95.8	245569.4	1.16	C12H8	Polycyclic aromatic hydrocarbon	aromatic	0
29.88	Cyclooctane, methyl-	85	720095.9	3.40	C9H18	Cycloalkane	naphthenes	0

30.31	Acenaphthene	82	38131.5	0.18	C12H10	Polycyclic aromatic hydrocarbon	aromatic	0
32.24	9-Eicosene, (E)-	90.9	1331371	6.28	C20H40	Alkene	olefins	0
32.96	Fluorene	84.5	168463.9	0.79	C13H10	Polycyclic aromatic hydrocarbon	aromatic	0
33.78	Benzene, 1,1'-(1,3-propanediyl)bis-	80.1	58677.3	0.28	C15H16	Diaryl alkane	aromatic	0
35.19	3-Eicosene, (E)-	93.7	1380850	6.51	C20H40	Alkene	olefins	0
36.31	(E)-Stilbene	84.8	42061.9	0.20	C14H12	Diarylethene	aromatic	0
39	Hexadecyl octyl ether	93.8	1325852	6.25	C24H50O	Dialkyl ether	others	0.00282

Table S5. GC-MS analysis result of oil produced during catalytic pyrolysis of Fe(III) modified kaolin with PP with rate 1:4.

RT	Name	Match, %	Area	ω, %	Formula	Category	Group	Weight content
4.08	Propene	95.4	50284.5	0.24	C3H6	Alkene	olefins	0
4.66	Water	92.5	415709.9	1.96	H2O	Inorganic solvent	others	0.01739
4.93	1-Propene, 2-methyl-	96.1	219754.7	1.03	C4H8	Alkene	olefins	0
4.98	1,2,4,5-Tetroxane, 3,3,6,6-tetramethyl-	86	74734	0.35	C8H16O4	Organic peroxide	others	0.00128
5.09	Cyclobutene	96.3	102827.9	0.48	C4H6	Cycloalkene	cyclic olefins	0
5.2	Oxetane, 3,3-dimethyl-	89.5	52610.8	0.25	C6H12O	Cyclic ether	others	0.0004
5.45	3,4-Dimethyldihydrofuran-2,5-dione	89.4	45877.2	0.22	C6H10O3	Cyclic anhydride	others	0.0008
6.82	1-Pentene	94.6	317726.5	1.50	C5H10	Alkene	olefins	0
6.97	Pentane	96.3	206209	0.97	C5H12	Alkane	parafins	0
7.28	Oxetane, 3-(1-methylethyl)-	85.3	53749.1	0.25	C6H12O	Cyclic ether	others	0.0004
10.19	1-Penten-3-one	80	77956.8	0.37	C5H8O	Î±,Î²â€‘Unsaturated ketone	ketones	0.0007
10.53	1-Penten-3-one	80.5	27463.7	0.13	C5H8O	Î±,Î²â€‘Unsaturated ketone	ketones	0.00025
12.54	Benzene	99.1	1124057.1	5.29	C6H6	Aromatic hydrocarbon	aromatic	0
12.77	1-Heptene	92.6	335302.4	1.58	C7H14	Alkene	olefins	0
12.91	Heptane	96.8	216448.3	1.02	C7H16	Alkane	parafins	0
13.87	Furan, 2-methoxy-	81.6	31828.9	0.15	C5H6O2	Heteroâ€‘aromatic ether	aromatic	0.00049
15.42	Cyclopropane, pentyl-	90.1	179205.4	0.84	C8H16	Cycloalkane	naphthenes	0
15.43	Toluene	97.5	458684.9	2.16	C7H8	Aromatic hydrocarbon	aromatic	0
15.54	Octane	96.2	195757.1	0.92	C8H18	Alkane	parafins	0
17.53	3-Heptyne, 5-methyl-	80.2	24540.8	0.12	C8H14	Alkyne	olefins	0
17.68	Ethylbenzene	92.8	204589	0.96	C8H10	Aromatic hydrocarbon	aromatic	0
17.71	1-Nonene	97.3	331909.6	1.56	C9H18	Alkene	olefins	0
17.81	Nonane	97.6	270595.9	1.27	C9H20	Alkane	parafins	0
17.85	Benzene, 1,3-dimethyl-	85.8	54255.1	0.26	C8H10	Aromatic hydrocarbon	aromatic	0
18.47	Styrene	98.8	1068677.7	5.03	C8H8	Vinylâ€‘aromatic	aromatic	0
19.73	1-Decene	97.1	590036	2.78	C10H20	Alkene	olefins	0

19.81	Decane	97.3	324214.1	1.53	C10H22	Alkane	parafins	0
20.23	.alpha.-Methylstyrene	91	109424	0.52	C9H10	Vinylâ€'aromatic	aromatic	0
20.56	Pimelic acid, di(3-phenylpropyl) ester	85.6	36065.1	0.17	C25H28O4	Diester	esters	0.00028
21.18	Diglycolic acid, di(3-phenylpropyl) ester	84.5	28032.5	0.13	C22H22O5	Diester	esters	0.00029
21.56	1-Undecene	98	577955.5	2.72	C11H22	Alkene	olefins	0
21.63	Undecane	97	409396.3	1.93	C11H24	Alkane	parafins	0
21.82	Benzene, 1-propynyl-	93.7	147702	0.70	C9H8	Aromatic hydrocarbon	aromatic	0
23.23	1-Dodecene	98	537123.2	2.53	C12H24	Alkene	olefins	0
23.29	Dodecane	92.7	399055.4	1.88	C12H26	Alkane	parafins	0
23.65	2-Methylindene	87.7	65190.6	0.31	C10H10	Polycyclic aromatic hydrocarbon	aromatic	0
23.81	Naphthalene, 1,2-dihydro-	85.4	51302.6	0.24	C10H12	Partially hydrogenated PAH	aromatic	0
24.56	Naphthalene	99.3	1557425	7.33	C10H8	Polycyclic aromatic hydrocarbon	aromatic	0
24.78	1-Tridecene	97.1	645428	3.04	C13H26	Alkene	olefins	0
24.83	Tridecane	90.4	367748.3	1.73	C13H28	Alkane	parafins	0
25.72	Hexadecane	87.4	976139.3	4.60	C16H34	Alkane	parafins	0
25.96	Naphthalene, 1-methyl-	84.5	20197.3	0.10	C11H10	Alkylâ€'PAH	aromatic	0
26.3	Cyclotetradecane	91.9	876900.7	4.13	C14H28	Cycloalkane	naphthenes	0
26.35	1H-Indene, 1-ethylidene-	85.4	143797	0.68	C11H10	Polycyclic aromatic hydrocarbon	aromatic	0
26.69	Naphthalene, 2-methyl-	88.9	106273	0.50	C11H10	Alkylâ€'PAH	aromatic	0
27.62	Biphenyl	96	353075.1	1.66	C12H10	Aromatic hydrocarbon	aromatic	0
27.95	Cyclooctane, methyl-	87.9	766143.9	3.61	C9H18	Cycloalkane	naphthenes	0
28.89	Naphthalene, 2-ethenyl-	87.5	85438.5	0.40	C12H10	Vinylâ€'PAH	aromatic	0
29.78	Acenaphthylene	94.9	197134.8	0.93	C12H8	Polycyclic aromatic hydrocarbon	aromatic	0
29.87	Cyclododecane	87.6	892688.3	4.20	C12H24	Cycloalkane	naphthenes	0
30.31	Acenaphthene	82	28454.9	0.13	C12H10	Polycyclic aromatic hydrocarbon	aromatic	0
32.22	Cycloheptane, methyl-	83.4	723865.3	3.41	C8H16	Cycloalkane	naphthenes	0
32.96	1H-Phenalene	85	94301.4	0.44	C13H10	Polycyclic aromatic hydrocarbon	aromatic	0
35.17	Cyclooctane, methyl-	80.2	822254.3	3.87	C9H18	Cycloalkane	naphthenes	0
35.19	1-Decanol, 2-hexyl-	94.9	1818746.1	8.56	C16H34O	Alcohol	alcohols	0.00565
36.3	Anthracene, 9,10-dihydro-9-(1-methylpropyl)-	83.7	24777.2	0.12	C18H20	Polycyclic aromatic hydrocarbon	aromatic	0
38.99	Oxalic acid, allyl octadecyl ester	93.5	1318262.7	6.21	C23H42O4	Monoâ€'ester	esters	0.01038

Table S6. GC-MS analysis result of oil produced during catalytic pyrolysis of Fe(III) modified kaolin with LDPE with rate 1:1.

RT	Name	Match, %	Area	ω, %	Formula	Category	Group	Weight content

12.54	Benzene	99.3	1452695	10.7	C6H6	Aromatic hydrocarbon	aromatic	0
35.19	Dodecyl nonyl ether	90.8	1194157	8.79	C21H44O	Dialkyl ether	others	0.004499
24.56	Naphthalene	99.3	1158079	8.53	C10H8	Polycyclic aromatic hydrocarbon	aromatic	0
29.88	Hexadecyl octyl ether	92.5	839045	6.18	C24H50O	Dialkyl ether	others	0.002788
32.24	Decyl octyl ether	93.5	810434.7	5.97	C18H38O	Dialkyl ether	others	0.003531
38.99	Decyl octyl ether	93.4	755057.3	5.56	C18H38O	Dialkyl ether	others	0.003289
25.71	Oxalic acid, allyl hexadecyl ester	80.3	512860.1	3.78	C21H38O4	Diâ€‘ester	esters	0.006823
4.66	Water	92.5	446975.6	3.29	H2O	Inorganic solvent	others	0.029218
38.98	Cycloheptane, methyl-	84.5	397682.7	2.93	C8H16	Cycloalkane	naphthenes	0
26.29	Cyclooctane, 1,4-dimethyl-, cis-	92.7	375817.1	2.77	C10H20	Cycloalkane	naphthenes	0
32.22	Cyclooctane, methyl-	85.3	355917	2.62	C9H18	Cycloalkane	naphthenes	0
27.95	Cycloheptane, methyl-	85.9	348832.3	2.57	C8H16	Cycloalkane	naphthenes	0
29.87	Cycloheptane, methyl-	81.9	346953.6	2.56	C8H16	Cycloalkane	naphthenes	0
6.82	1-Pentene	96.3	271027.7	2	C5H10	Alkene	olefins	0
27.62	Biphenyl	96.4	260783.3	1.92	C12H10	Aromatic hydrocarbon	aromatic	0
24.78	Cyclopropane, nonyl-	88.9	255838	1.88	C12H24	Cycloalkane	naphthenes	0
4.92	1-Propene, 2-methyl-	94.5	251117.7	1.85	C4H8	Alkene	olefins	0
32.67	1H-Indene, 1-(phenylmethylene)-	83.6	206139.6	1.52	C16H12	Polycyclic aromatic hydrocarbon	aromatic	0
32.96	Fluorene	85.3	202741.6	1.49	C13H10	Polycyclic aromatic hydrocarbon	aromatic	0
6.97	Pentane	95.2	197466.3	1.45	C5H12	Alkane	parafins	0
24.83	Heptane, 2,5,5-trimethyl-	82.4	195946	1.44	C10H22	Branched alkane	parafins	0
23.23	Cyclopropane, octyl-	95.8	183801.2	1.35	C11H22	Cycloalkane	naphthenes	0
23.29	Octane, 2,5,6-trimethyl-	82.8	166147.8	1.22	C11H24	Branched alkane	parafins	0
29.78	Acenaphthylene	94.3	163571	1.2	C12H8	Polycyclic aromatic hydrocarbon	aromatic	0
12.76	1-Heptene	91.2	159177	1.17	C7H14	Alkene	olefins	0
12.91	Heptane	96.3	145055.7	1.07	C7H16	Alkane	parafins	0
26.35	Naphthalene, 2-methyl-	88.8	132748	0.98	C11H10	Alkylâ€‘PAH	aromatic	0
16.38	Propane, 2,2'-[ethylidenebis(oxy)]bis-	95.3	122442.3	0.9	C8H18O2	Diether / acetal	others	0.001969
21.56	1-Octene, 3,7-dimethyl-	94.3	122396.5	0.9	C10H20	Branched alkene	olefins	0
5.09	1,3-Butadiene	96.3	122209.3	0.9	C4H6	Conjugated diene	olefins	0
26.69	1H-Indene, 1-ethylidene-	88.1	109270.1	0.8	C11H10	Polycyclic aromatic hydrocarbon	aromatic	0
24.13	1H-Indene, 1-(phenylmethylene)-	84.7	93668.5	0.69	C16H12	Polycyclic aromatic hydrocarbon	aromatic	0
21.63	Pentane, 2,2,3,4-tetramethyl-	89.1	90547.7	0.67	C9H20	Branched alkane	parafins	0
10.18	2-Hexene, (E)-	85.9	89667.8	0.66	C6H12	Alkene	olefins	0
15.43	Toluene	90.6	88535.9	0.65	C7H8	Aromatic hydrocarbon	aromatic	0
4.98	1,2,4,5-Tetroxane, 3,3,6,6-tetramethyl-	82.8	80414.5	0.59	C8H16O4	Organic peroxide	others	0.002143

7.28	Oxetane, 3-(1-methylethyl)-	91.2	74153.4	0.55	C6H12O	Cyclic ether	others	0.000879
21.82	Benzene, 1-ethynyl-4-methyl-	88.8	71538.6	0.53	C9H8	Aromatic hydrocarbon	aromatic	0
5.45	1-Propene, 2-methyl-	91.9	68289.5	0.5	C4H8	Alkene	olefins	0
10.08	2-Pentene, 3-methyl-, (Z)-	87.5	65391.2	0.48	C6H12	Alkene	olefins	0
5.19	3,4-Dimethyldihydrofuran-2,5-dione	90	60118	0.44	C6H10O3	Cyclic anhydride	others	0.001623
4.08	Propene	95.3	59083.2	0.44	C3H6	Alkene	olefins	0
28.89	Biphenyl	86	56909.9	0.42	C12H10	Aromatic hydrocarbon	aromatic	0
18.47	Nicotinic acid, 2-phenylethyl ester	85.5	54036.5	0.4	C14H13NO2	Aromatic ester	esters	0.000563
10.7	2-Pentene, 2-methyl-	80.3	53622.5	0.39	C6H12	Alkene	olefins	0
7.47	Cyclopentene	84.5	53587.6	0.39	C5H8	Cycloalkene	cyclic olefins	0
19.73	1-Octene, 3,7-dimethyl-	87.8	53521.5	0.39	C10H20	Branched alkene	olefins	0
23.64	2-Methylindene	82.2	48710.1	0.36	C10H10	Polycyclic aromatic hydrocarbon	aromatic	0
23.81	Naphthalene, 1,2-dihydro-	81.7	45473.4	0.33	C10H12	Partially hydrogenated PAH	aromatic	0
10.53	3-Hexene, (E)-	90.8	44463.8	0.33	C6H12	Alkene	olefins	0
12.73	1,1'-Bicyclobutyl	80.4	31094.5	0.23	C8H12	Bicyclic cycloalkane	naphthenes	0
10.41	2-Pentene, 4-methyl-	80.9	18848.8	0.14	C6H12	Alkene	olefins	0
20.57	Benzeneethanol, .beta.-ethenyl-	82.2	14974.1	0.11	C9H10O	Aromatic alcohol	alcohols	0.000131

Table S7. GC-MS analysis result of oil produced during catalytic pyrolysis of Fe(III) modified kaolin with LDPE with rate 1:2.

RT	Name	Match, %	Area	ω, %	Formula	Category	Group	Oxygen content
4.66	Water	92.5	442721	3.14	H2O	Inorganic solvent	others	0.028
4.93	2-Butene, (Z)-	95.7	323944	2.30	C4H8	Alkene	olefins	0.000
4.99	1,2,4,5-Tetroxane, 3,3,6,6-tetramethyl-	89.7	113853.8	0.81	C8H16O4	Organic peroxide	others	0.003
5.1	Cyclobutene	95.5	140975.2	1.00	C4H6	Cycloalkene	cyclic olefins	0.000
5.2	2-Butene, (Z)-	92.3	82749.2	0.59	C4H8	Alkene	olefins	0.000
5.46	1-Propene, 2-methyl-	90.8	73740.4	0.52	C4H8	Alkene	olefins	0.000
6.83	1-Pentene	95.7	386244.9	2.74	C5H10	Alkene	olefins	0.000
6.97	Pentane	93.6	271076.8	1.92	C5H12	Alkane	parafins	0.000
7.28	Oxetane, 3-(1-methylethyl)-	91.1	89364.4	0.63	C7H14O	Cyclic ether	others	0.001
7.48	1,4-Pentadiene	80.4	52003	0.37	C5H8	Diene (alkene)	olefins	0.000
10.08	2-Butene, 2,3-dimethyl-	85.4	43470.9	0.31	C6H12	Alkene	olefins	0.000
10.19	2-Hexene	88.9	88789.6	0.63	C6H12	Alkene	olefins	0.000
10.53	3-Hexene	87.3	44201.8	0.31	C6H12	Alkene	olefins	0.000
10.7	Cyclopropane, 1,1,2-trimethyl-	82.4	37915.9	0.27	C6H12	Cycloalkane	naphthenes	0.000
11.59	1,3-Cyclopentadiene, 1-methyl-	83.4	51462.9	0.36	C6H8	Cyclodiene	cyclic olefins	0.000

12.54	Benzene	99.1	1395555.3	9.89	C6H6	Aromatic hydrocarbon	aromatic	0.000
12.73	7-Azabicyclo[4,2,0]octan-8-one	80.3	41488.1	0.29	C7H11NO	Bicyclic ketone	ketones	0.000
12.77	1-Heptene	92.4	206918.8	1.47	C7H14	Alkene	olefins	0.000
12.92	Heptane	96	169908.3	1.20	C7H16	Alkane	parafins	0.000
15.42	Isopropylcyclobutane	86.3	49389	0.35	C7H14	Cycloalkane	naphthenes	0.000
15.43	Toluene	97.7	319287.4	2.26	C7H8	Aromatic hydrocarbon	aromatic	0.000
15.54	Hexane, 3,3-dimethyl-	82.4	39253.1	0.28	C8H18	Branched alkane	parafins	0.000
17.68	Ethylbenzene	88.4	68120.2	0.48	C8H10	Aromatic hydrocarbon	aromatic	0.000
17.71	Cyclobutane, butyl-	87.3	68131.6	0.48	C8H16	Cycloalkane	naphthenes	0.000
17.81	Hexane, 2,4-dimethyl-	90.2	68531	0.49	C8H18	Branched alkane	parafins	0.000
17.85	Benzene, 1,3-dimethyl-	87.6	61098.6	0.43	C8H10	Aromatic hydrocarbon	aromatic	0.000
18.47	Styrene	91.3	326107.1	2.31	C8H8	Vinylâ€'aromatic	aromatic	0.000
19.73	Cyclopropane, 1-hexyl-2-methyl-	95.2	224283.3	1.59	C10H20	Cycloalkane	naphthenes	0.000
19.82	Decane	92.3	131293	0.93	C10H22	Alkane	parafins	0.000
20.22	Azetidine, 3-methyl-3-phenyl-	89.8	20760.7	0.15	C10H11N	Heterocycle (amine)	others	0.000
20.57	Pimelic acid, di(3-phenylpropyl) ester	85.2	38648.3	0.27	C25H28O4	Diester	esters	0.000
21.56	Cyclopropane, octyl-	97	280593.8	1.99	C11H22	Cycloalkane	naphthenes	0.000
21.63	Undecane	95.2	199981.2	1.42	C11H24	Alkane	parafins	0.000
21.83	Benzene, 1-ethynyl-4-methyl-	91.1	120739.3	0.86	C9H8	Aromatic hydrocarbon	aromatic	0.000
23.23	1-Dodecene	97.1	294967.4	2.09	C12H24	Alkene	olefins	0.000
23.29	Dodecane	86.1	236579.1	1.68	C12H26	Alkane	parafins	0.000
23.65	2-Methylindene	90.2	64259.3	0.46	C10H10	Polycyclic aromatic hydrocarbon	aromatic	0.000
23.81	Naphthalene, 1,2-dihydro-	88.5	55629.7	0.39	C10H12	Partially hydrogenated PAH	aromatic	0.000
24.56	Naphthalene	99	1033943.7	7.33	C10H8	Polycyclic aromatic hydrocarbon	aromatic	0.000
24.78	1-Tridecene	93.2	343979	2.44	C13H26	Alkene	olefins	0.000
24.83	Nonane, 3,7-dimethyl-	84.6	163212.2	1.16	C11H24	Branched alkane	parafins	0.000
25.74	Dodecane, 1-iodo-	88.4	781164.7	5.54	C12H25I	Alkyl iodide	others	0.000
26.3	7-Tetradecene, (Z)-	92.9	658971.8	4.67	C14H28	Alkene	olefins	0.000
26.35	1H-Indene, 1-ethylidene-	86.8	100651.9	0.71	C11H10	Polycyclic aromatic hydrocarbon	aromatic	0.000
26.69	Naphthalene, 2-methyl-	86.5	74770	0.53	C11H10	Alkylâ€'PAH	aromatic	0.000
27.63	Biphenyl	95.4	192747.5	1.37	C12H10	Aromatic hydrocarbon	aromatic	0.000
27.95	1-Pentadecene	92.6	747106	5.29	C15H30	Alkene	olefins	0.000
28.89	Acenaphthene	81.6	38557.2	0.27	C12H10	Polycyclic aromatic hydrocarbon	aromatic	0.000
29.78	Acenaphthylene	91.7	98921.1	0.70	C12H8	Polycyclic aromatic hydrocarbon	aromatic	0.000

29.88	Cycloheptane, methyl-	83	272990.5	1.93	C8H16	Cycloalkane	naphthenes	0.000
29.92	Oxalic acid, allyl hexadecyl ester	90.7	692627.3	4.91	C21H38O4	Monoâ€'ester	esters	0.009
32.23	Oxalic acid, isobutyl hexadecyl ester	91.5	585330	4.15	C22H42O4	Monoâ€'ester	esters	0.007
32.68	Naphthalene, 1-phenyl-	81.8	103554.5	0.73	C16H10	Polycyclic aromatic hydrocarbon	aromatic	0.000
32.97	1H-Phenalene	82.3	66041.2	0.47	C13H10	Polycyclic aromatic hydrocarbon	aromatic	0.000
35.18	Cyclooctane, methyl-	85.8	296282.7	2.10	C9H18	Cycloalkane	naphthenes	0.000
35.2	Heptyl tetradecyl ether	91.7	685091.5	4.85	C21H44O	Dialkyl ether	others	0.002
39	Oxalic acid, allyl pentadecyl ester	86.9	451246.5	3.20	C20H36O4	Monoâ€'ester	esters	0.006

Table S8. GC-MS analysis result of oil produced during catalytic pyrolysis of Fe(III) modified kaolin with LDPE with rate 1:4.

RT	Name	Match, %	Area	ω, %	Formula	Category	Group	Oxygen content
4.08	Propene	93.4	86178.4	0.48	C3H6	Alkene	olefins	0.000
4.67	Water	92.5	255220	1.41	H2O	Inorganic solvent	others	0.012
4.93	1-Propene, 2-methyl-	98.1	459134.6	2.53	C4H8	Alkene	olefins	0.000
4.98	1,2,4,5-Tetroxane, 3,3,6,6-tetramethyl-	90.6	144809.3	0.80	C8H16O4	Organic peroxide	others	0.003
5.09	1,3-Butadiene	97.4	197299.7	1.09	C4H6	Conjugated diene	olefins	0.000
5.2	2-Butene, (E)-	93.4	138413.8	0.76	C4H8	Alkene	olefins	0.000
5.45	1-Propene, 2-methyl-	92.6	84786.1	0.47	C4H8	Alkene	olefins	0.000
6.83	1-Pentene	96.7	561543.2	3.10	C5H10	Alkene	olefins	0.000
6.97	Pentane	95.3	325314.5	1.79	C5H12	Alkane	parafins	0.000
7.28	Oxetane, 3-(1-methylethyl)-	91.6	84656	0.47	C7H14O	Cyclic ether	others	0.001
7.48	Cyclobutane, methylene-	83	74938.9	0.41	C5H8	Cycloalkene	cyclic olefins	0.000
10.08	2-Butene, 2,3-dimethyl-	84.2	77569.5	0.43	C6H12	Alkene	olefins	0.000
11.6	4-Methylenecyclopentene	88.5	65105.8	0.36	C6H8	Cycloalkene	cyclic olefins	0.000
11.78	1,4-Cyclohexadiene	87.7	80927.4	0.45	C6H8	Cyclodiene	cyclic olefins	0.000
12.54	Benzene	98.9	1509798.3	8.32	C6H6	Aromatic hydrocarbon	aromatic	0.000
12.73	7-Azabicyclo[4,2,0]octan-8-one	84	57733.7	0.32	C7H11NO	Bicyclic ketone	ketones	0.000
12.77	1-Heptene	92.6	292157.9	1.61	C7H14	Alkene	olefins	0.000
12.91	Heptane	96.8	223965	1.23	C7H16	Alkane	parafins	0.000
13.87	Furan, 2-methoxy-	83.1	16272.7	0.09	C5H6O2	Hetero-aromatic ether	aromatic	0.000
15.41	Formic acid, hexyl ester	85.4	85127	0.47	C7H14O2	Ester	esters	0.001
15.43	Toluene	98.2	441422.3	2.43	C7H8	Aromatic hydrocarbon	aromatic	0.000
15.54	Octane	92.5	71626.4	0.39	C8H18	Alkane	parafins	0.000

17.68	Ethylbenzene	91	69719.5	0.38	C8H10	Aromatic hydrocarbon	aromatic	0.000
17.71	1-Nonene	91.3	133781.7	0.74	C9H18	Alkene	olefins	0.000
17.81	Nonane	92.7	108768.1	0.60	C9H20	Alkane	parafins	0.000
17.85	Benzene, 1,3-dimethyl-	92.4	59132.1	0.33	C8H10	Aromatic hydrocarbon	aromatic	0.000
18.47	Styrene	93.4	422269.5	2.33	C8H8	Vinyl-aromatic	aromatic	0.000
19.73	1-Decene	97.3	358877.7	1.98	C10H20	Alkene	olefins	0.000
19.81	Decane	94.2	188988.9	1.04	C10H22	Alkane	parafins	0.000
20.48	1,3-Benzenediol, monobenzoate	80.2	19108.2	0.11	C13H10O3	Aromatic ester	esters	0.000
21.56	Cyclopropane, octyl-	97.6	432800.8	2.39	C11H22	Cycloalkane	naphthenes	0.000
21.63	Undecane	96.2	276922	1.53	C11H24	Alkane	parafins	0.000
21.82	Indene	94.3	153521.5	0.85	C9H8	Polycyclic aromatic hydrocarbon	aromatic	0.000
23.23	Cyclopropane, octyl-	93.5	470422.8	2.59	C11H22	Cycloalkane	naphthenes	0.000
23.29	Undecane, 4,7-dimethyl-	93.9	311324.3	1.72	C13H28	Branched alkane	parafins	0.000
23.65	2-Methylindene	88.1	70698.8	0.39	C10H10	Polycyclic aromatic hydrocarbon	aromatic	0.000
24.01	Naphthalene, 1,2-dihydro-	84.2	40012.9	0.22	C10H10	Partially hydrogenated PAH	aromatic	0.000
24.56	Naphthalene	99.4	1388345.6	7.65	C10H8	Polycyclic aromatic hydrocarbon	aromatic	0.000
24.78	1-Tridecene	96.6	552453.4	3.05	C13H26	Alkene	olefins	0.000
24.83	Nonane, 3,7-dimethyl-	85.2	254940.8	1.41	C11H24	Branched alkane	parafins	0.000
25.77	Borane, diethyl(decyloxy)-	84.8	343084.6	1.89	C14H31BO3	Organoborane ether	others	0.004
25.95	Naphthalene, 2-methyl-	84.7	17315.2	0.10	C11H10	Alkyl-PAH	aromatic	0.000
26.3	Cyclotetradecane	94.8	752055.7	4.15	C14H28	Cycloalkane	naphthenes	0.000
26.35	Naphthalene, 1-methyl-	89.2	130232.4	0.72	C11H10	Alkyl-PAH	aromatic	0.000
26.7	1H-Indene, 1-ethylidene-	90.2	92323.2	0.51	C11H10	Polycyclic aromatic hydrocarbon	aromatic	0.000
27.62	Biphenyl	96.9	300443.8	1.66	C12H10	Aromatic hydrocarbon	aromatic	0.000
27.95	Cyclooctane, methyl-	85.8	650339.6	3.59	C9H18	Cycloalkane	naphthenes	0.000
28.88	Acenaphthene	86.5	50971.8	0.28	C12H10	Polycyclic aromatic hydrocarbon	aromatic	0.000
29.78	Acenaphthylene	93.7	142439.8	0.79	C12H8	Polycyclic aromatic hydrocarbon	aromatic	0.000
29.88	1-Dodecene	90.4	1220613.3	6.73	C12H24	Alkene	olefins	0.000
32.21	Cyclododecane	84.3	546928	3.02	C12H24	Cycloalkane	naphthenes	0.000
32.96	Fluorene	84.1	84993.8	0.47	C13H10	Polycyclic aromatic hydrocarbon	aromatic	0.000
35.18	Cyclopentane, 2-isopropyl-1,3-dimethyl-	82.4	721574.8	3.98	C11H22	Branched cycloalkane	naphthenes	0.000
35.2	3-Octadecene, (E)-	93.1	1348436.1	7.43	C18H36	Alkene	olefins	0.000
36.29	(E)-Stilbene	88.5	33877	0.19	C14H12	Diarylethene	aromatic	0.000
39	Oxalic acid, allyl octadecyl ester	91.8	1054884.3	5.82	C23H42O4	Mono-ester	esters	0.010

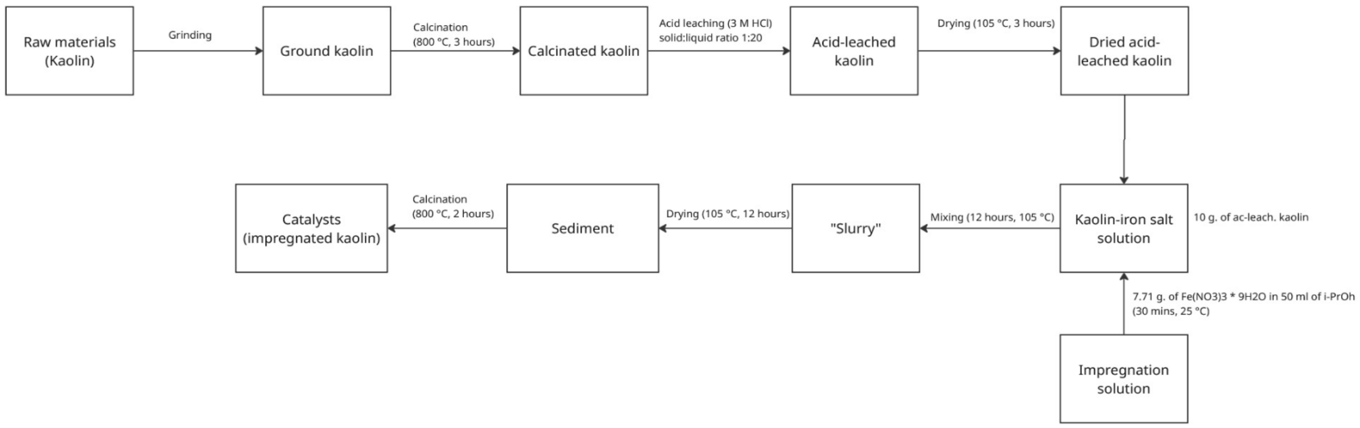

Figure S1. Catalytic material preparation procedure.

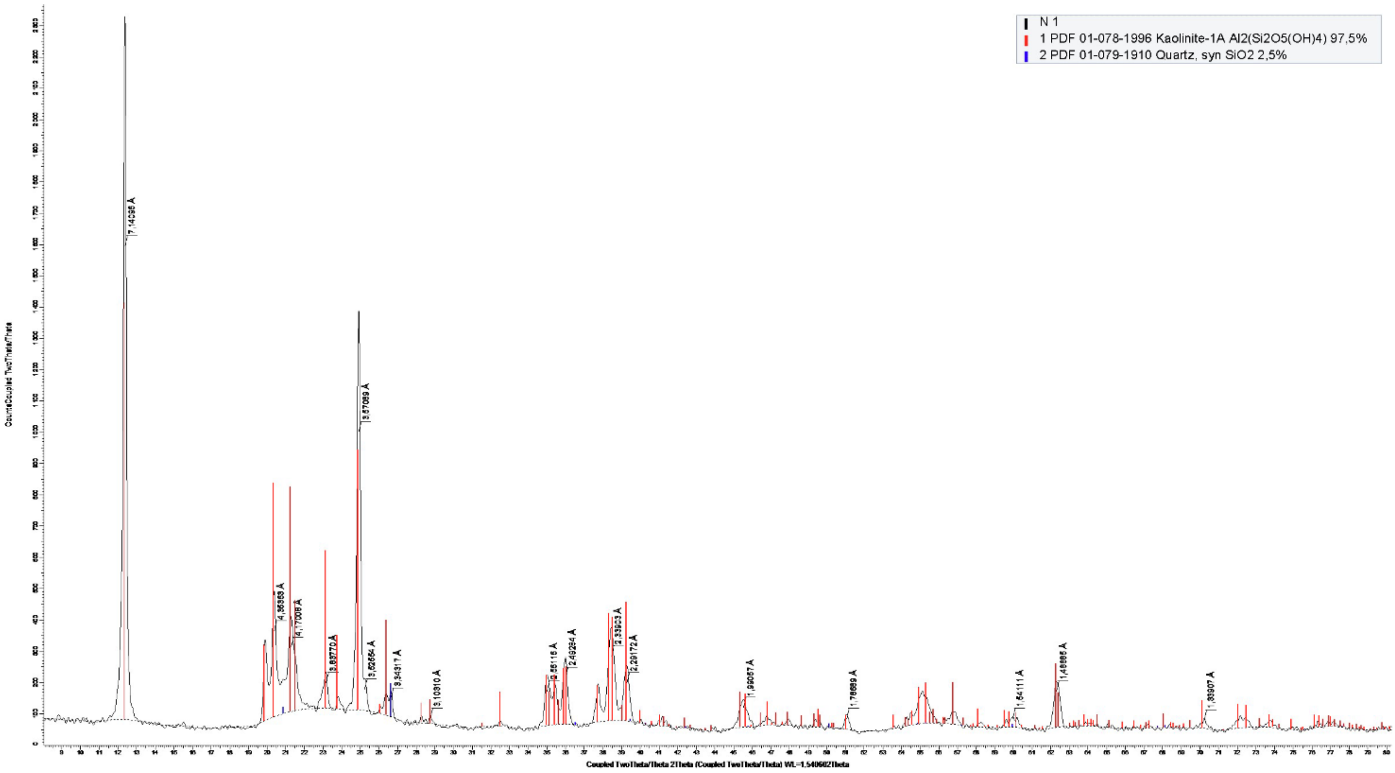

(a)

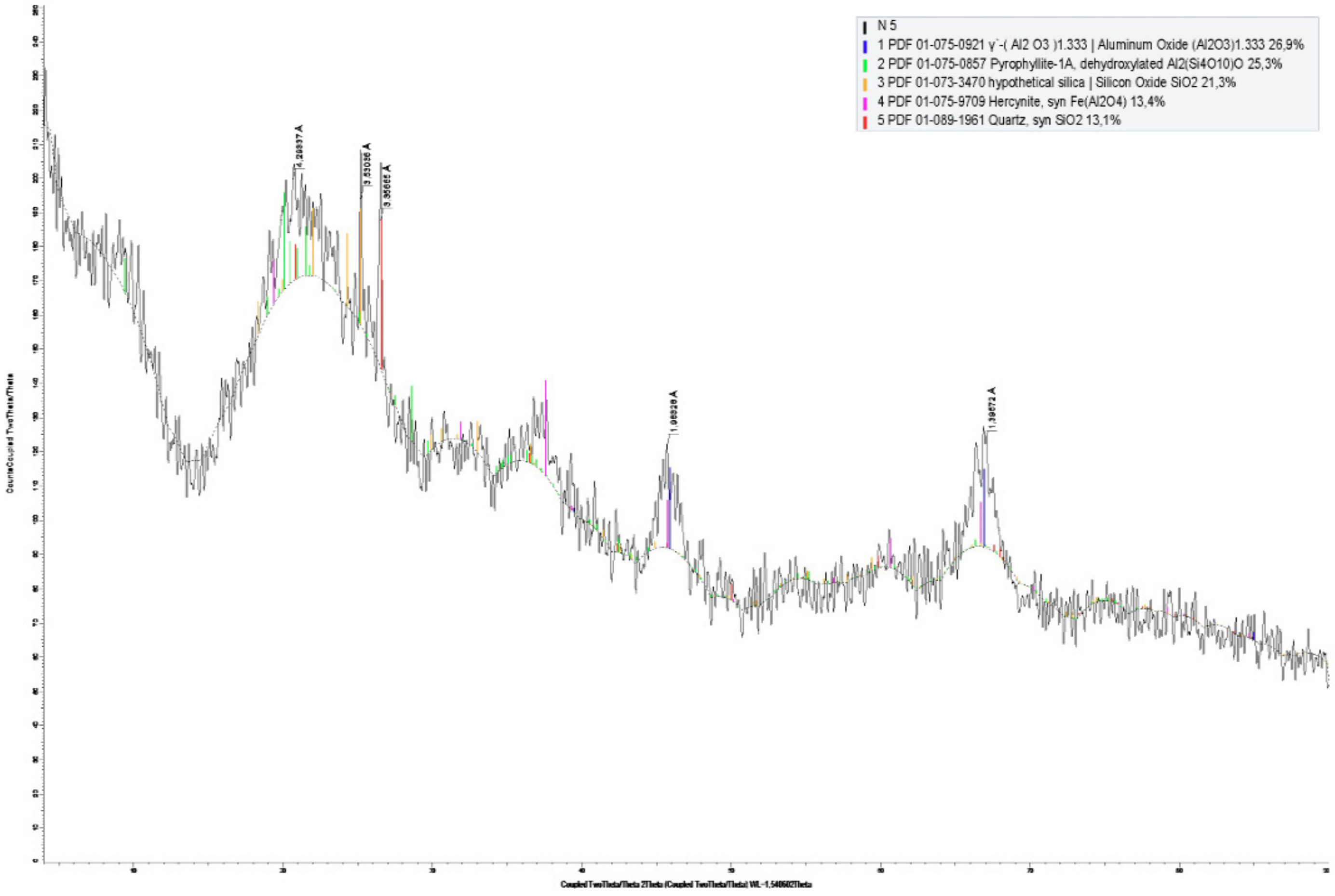
N 5
1 PDF 01-075-0921 γ`-(Al2 O3)1.333 | Aluminum Oxide (Al2O3)1.333 26,9%
2 PDF 01-075-0857 Pyrophyllite-1A, dehydroxylated Al2(Si4O10)O 25,3%
3 PDF 01-073-3470 hypothetical silica | Silicon Oxide SiO2 21,3%
4 PDF 01-075-9709 Hercynite, syn Fe(Al2O4) 13,4%
5 PDF 01-089-1961 Quartz, syn SiO2 13,1%

(b)

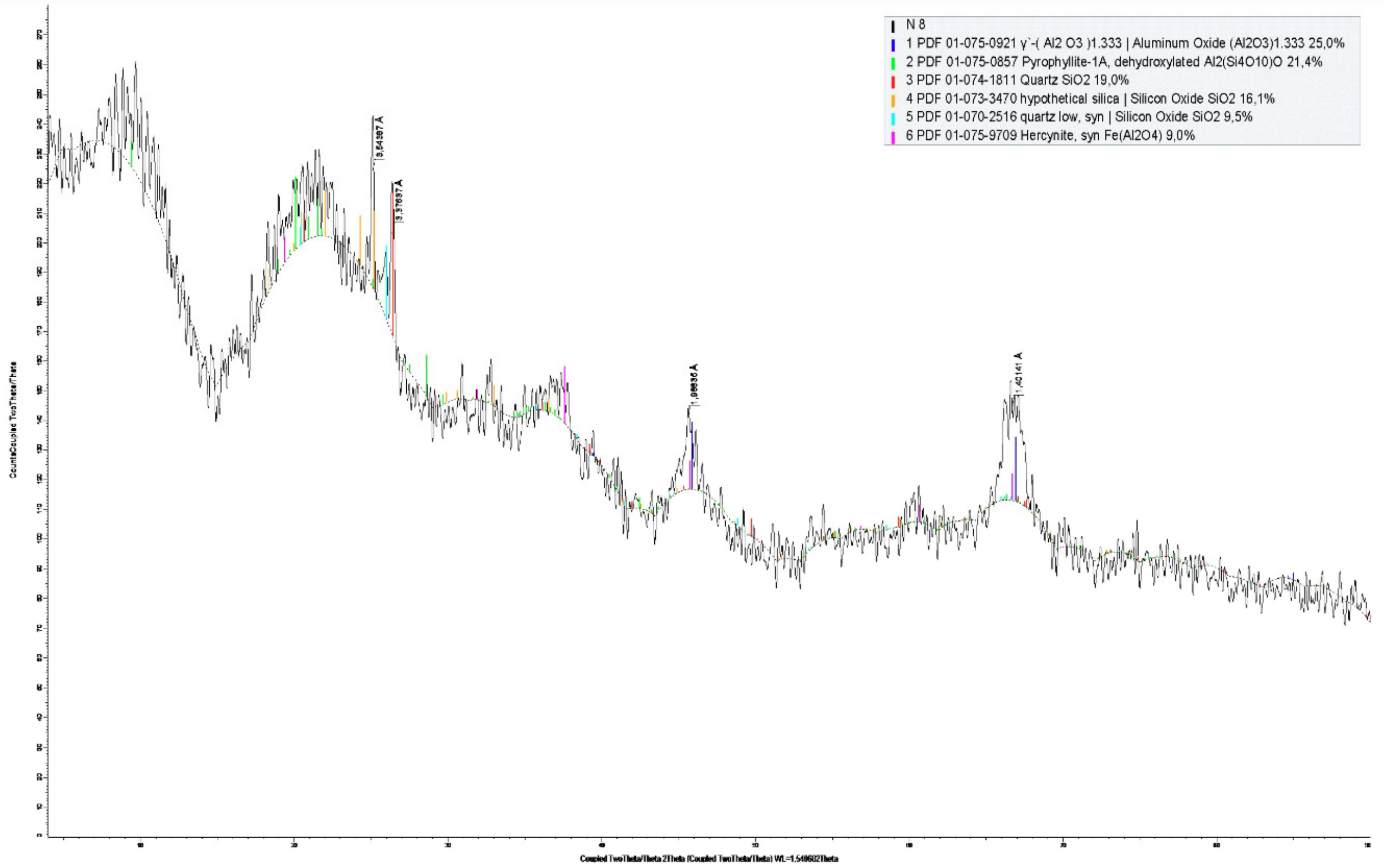
N 8
1 PDF 01-075-0921 γ`-(Al2 O3)1.333 | Aluminum Oxide (Al2O3)1.333 25,0%
2 PDF 01-075-0857 Pyrophyllite-1A, dehydroxylated Al2(Si4O10)O 21,4%
3 PDF 01-074-1811 Quartz SiO2 19,0%
4 PDF 01-073-3470 hypothetical silica | Silicon Oxide SiO2 16,1%
5 PDF 01-070-2516 quartz low, syn | Silicon Oxide SiO2 9,5%
6 PDF 01-075-9709 Hercynite, syn Fe(Al2O4) 9,0%

(c)

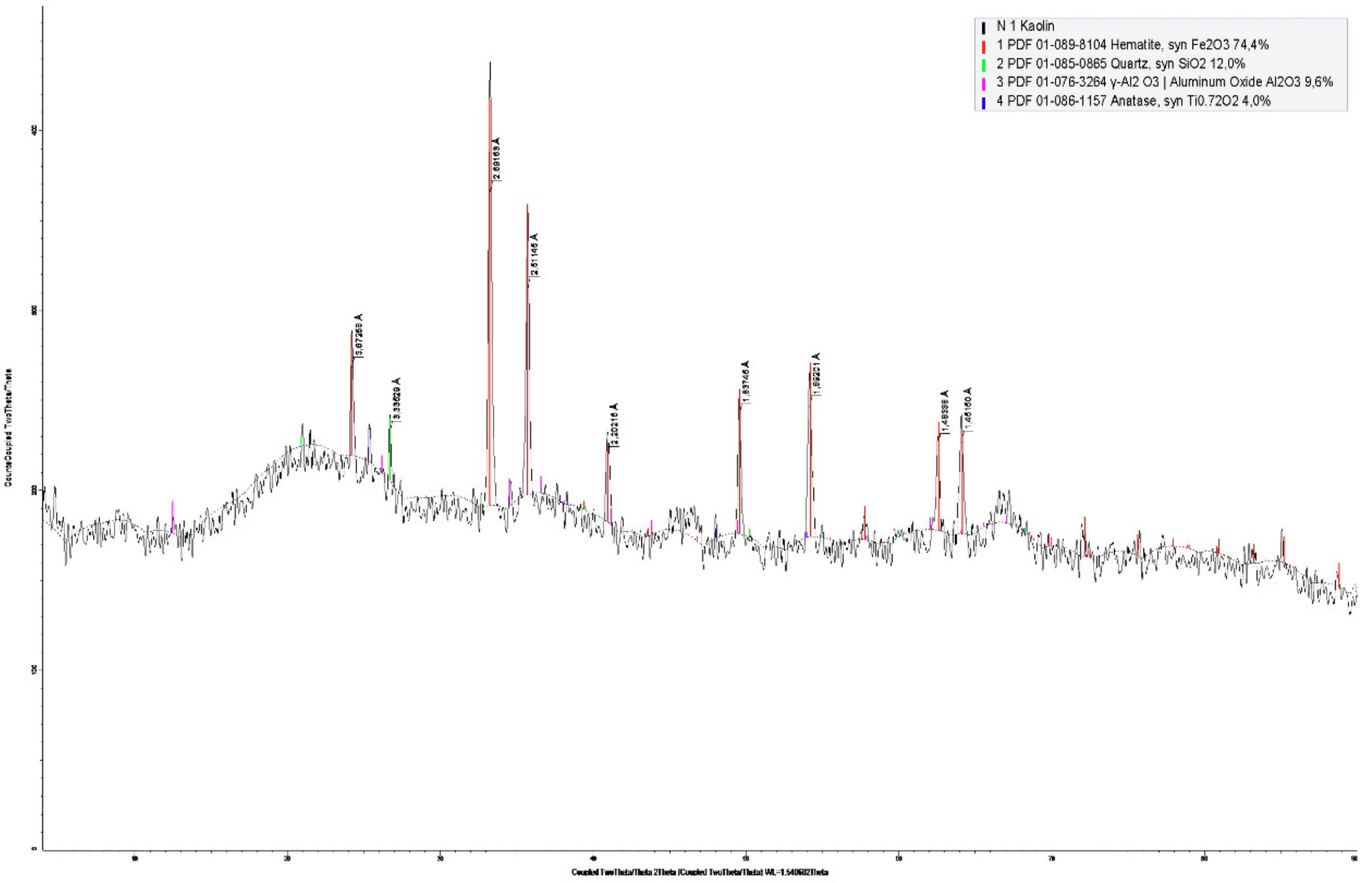

(d)

Figure S2. XRD analyses of catalytic materials: (a) Kaolin, as received, wt%; (b) Kaolin, calcined; (c) Kaolin, acid leached; (d) Kaolin, impregnated.

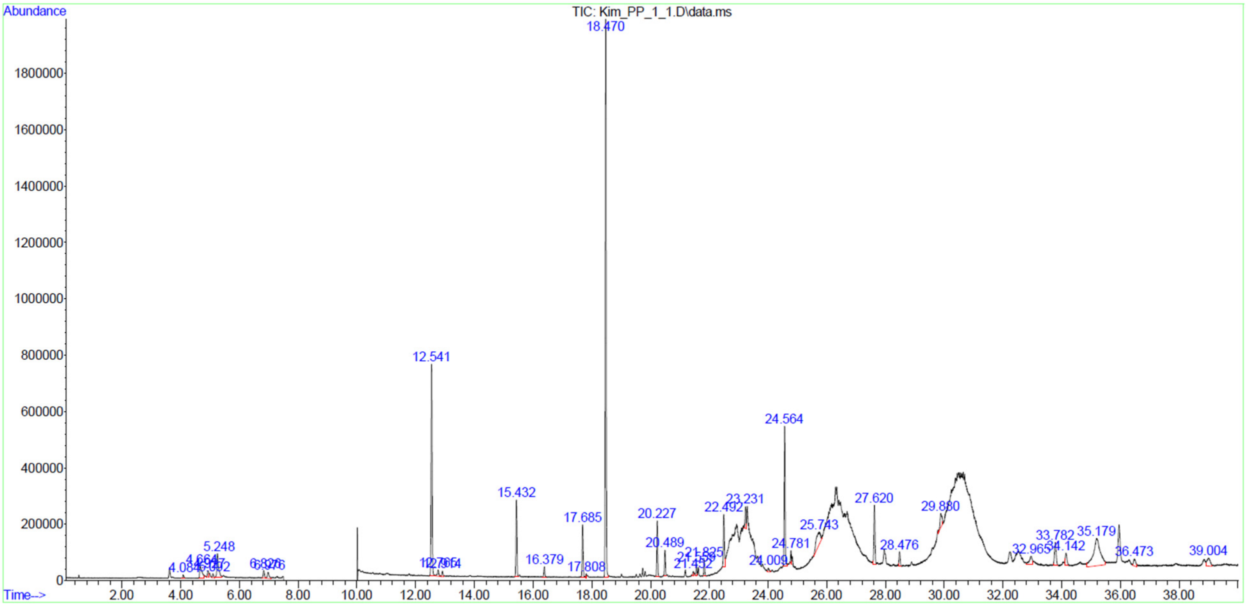

(a)

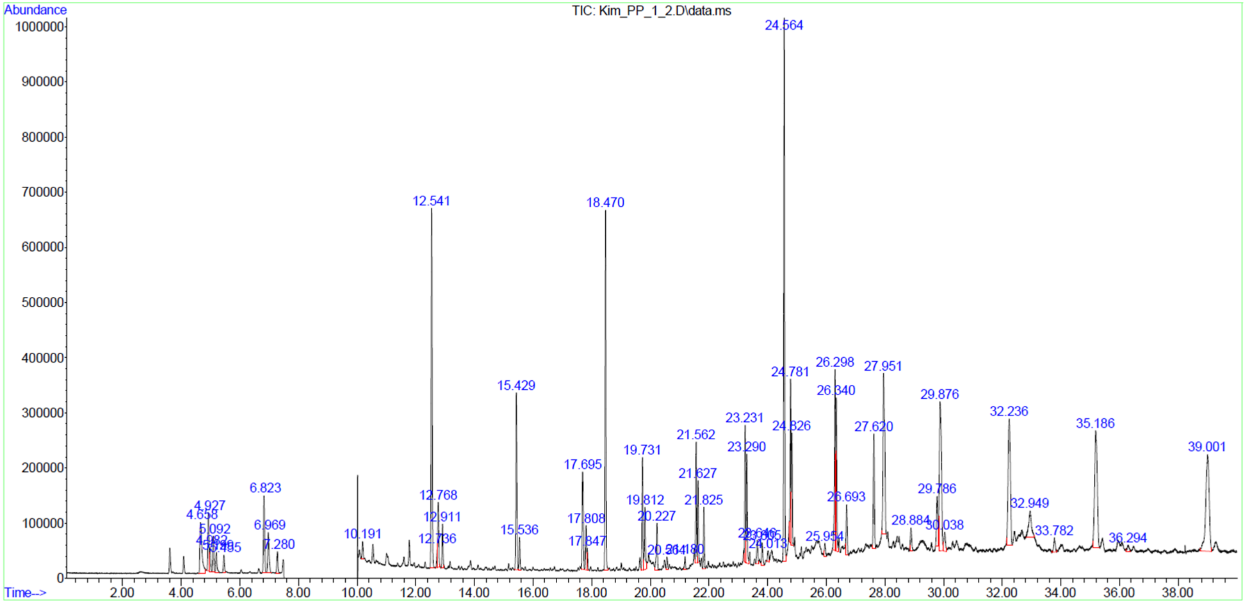
Abundance
TIC: Kim_PP_1_2.D\data.ms
Time-->
24.564
12.541
18.470
15.429
26.298
27.951
24.781
26.340
29.876
32.236
35.186
39.001
23.231
21.562
27.620
24.826
19.731
23.290
17.695
21.627
6.823
12.768
19.812
21.825
26.693
29.786
32.949
4.927
4.658
12.911
17.808
20.227
28.884
5.092
6.969
10.191
15.536
30.038
33.782
7.280
12.736
17.847
25.954
36.294

(b)

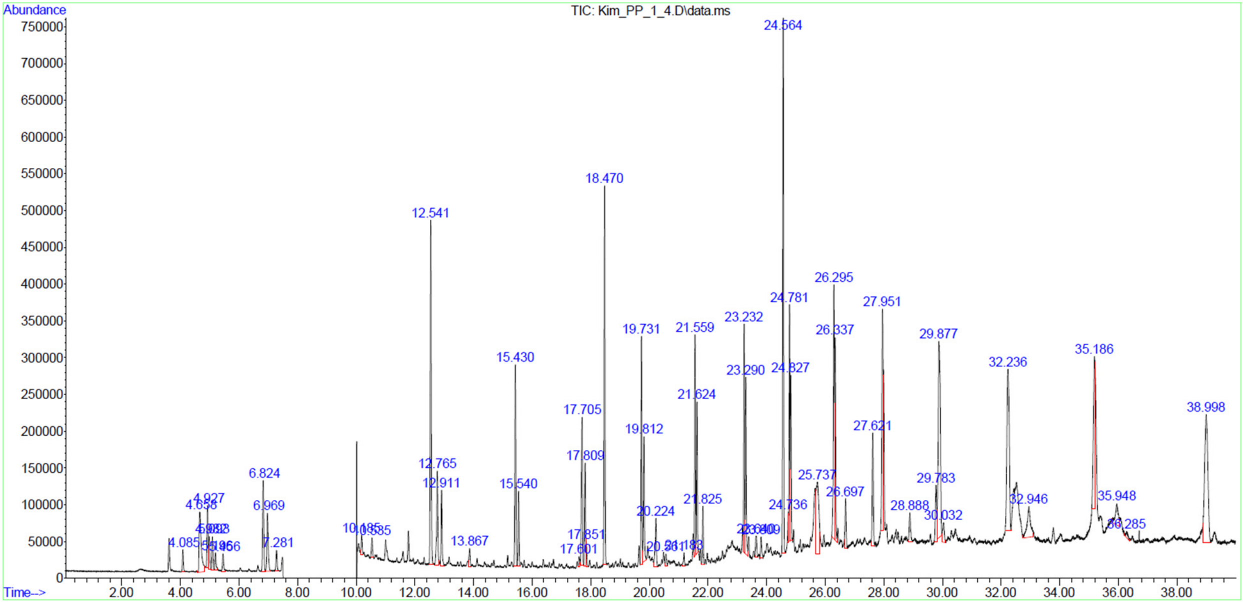
Abundance
TIC: Kim_PP_1_4.D\data.ms
Time-->
24.564
18.470
12.541
26.295
24.781
27.951
23.232
19.731
21.559
26.337
29.877
15.430
35.186
32.236
23.290
24.827
21.624
17.705
38.998
27.621
19.812
17.809
12.765
6.824
25.737
12.911
15.540
29.783
26.697
4.927
4.658
6.969
20.224
24.736
21.825
32.946
35.948
28.888
30.032
36.285
13.867
17.851
17.601
7.281
4.085

(c)

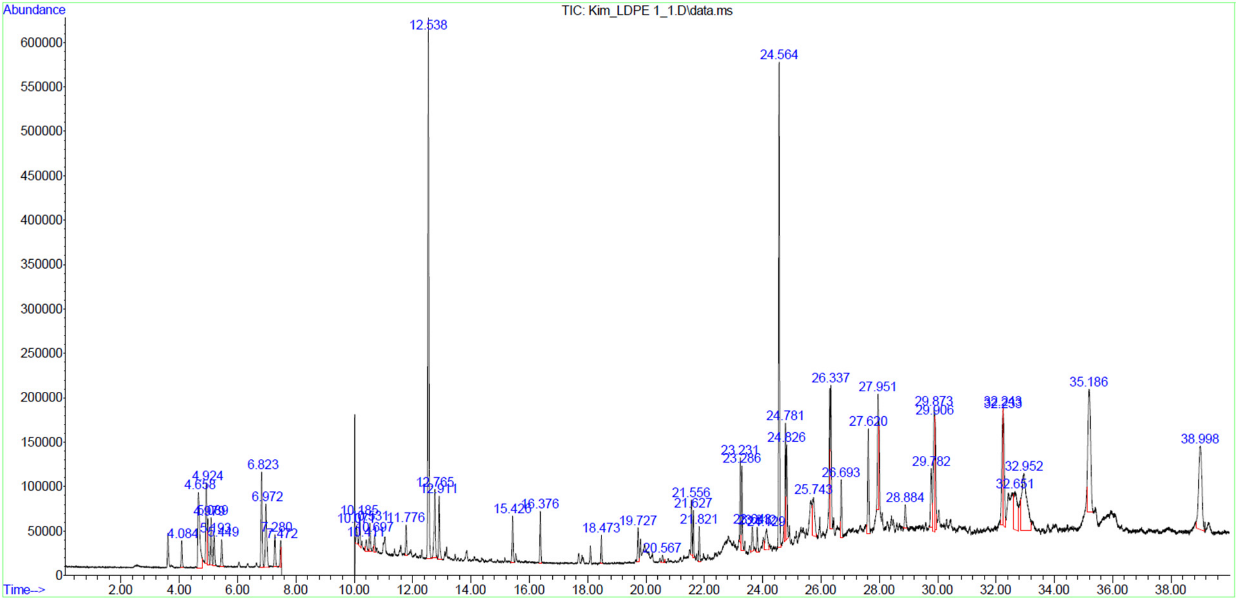
TIC: Kim_LDPE 1_1.D\data.ms
Abundance
Time-->

(d)

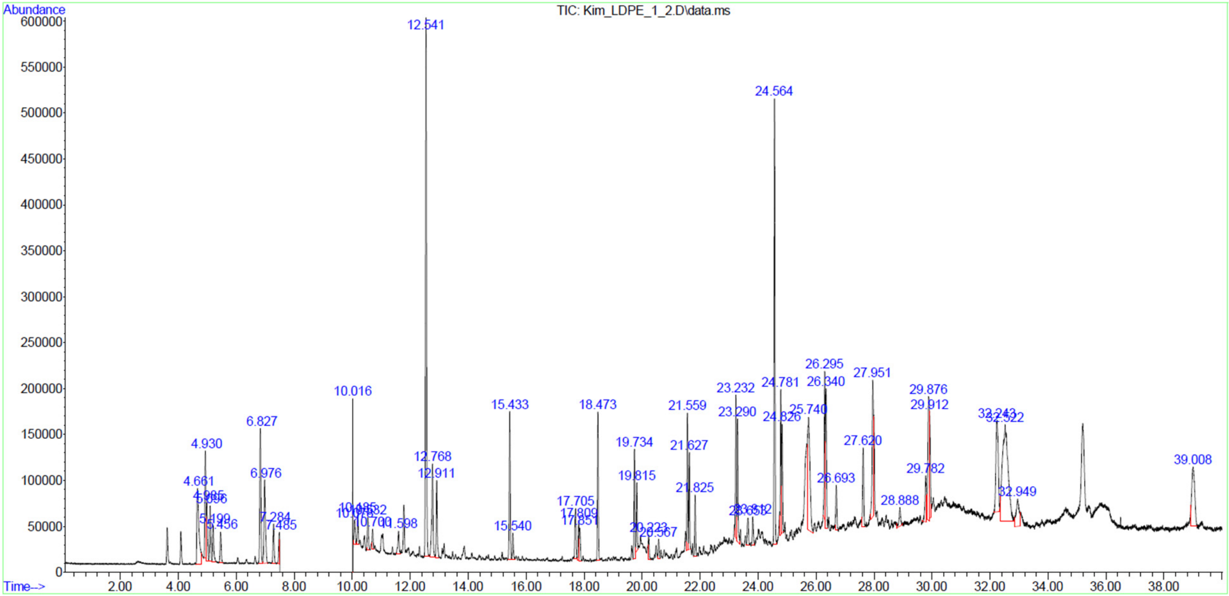
TIC: Kim_LDPE_1_2.D\data.ms
Abundance
Time-->

(e)

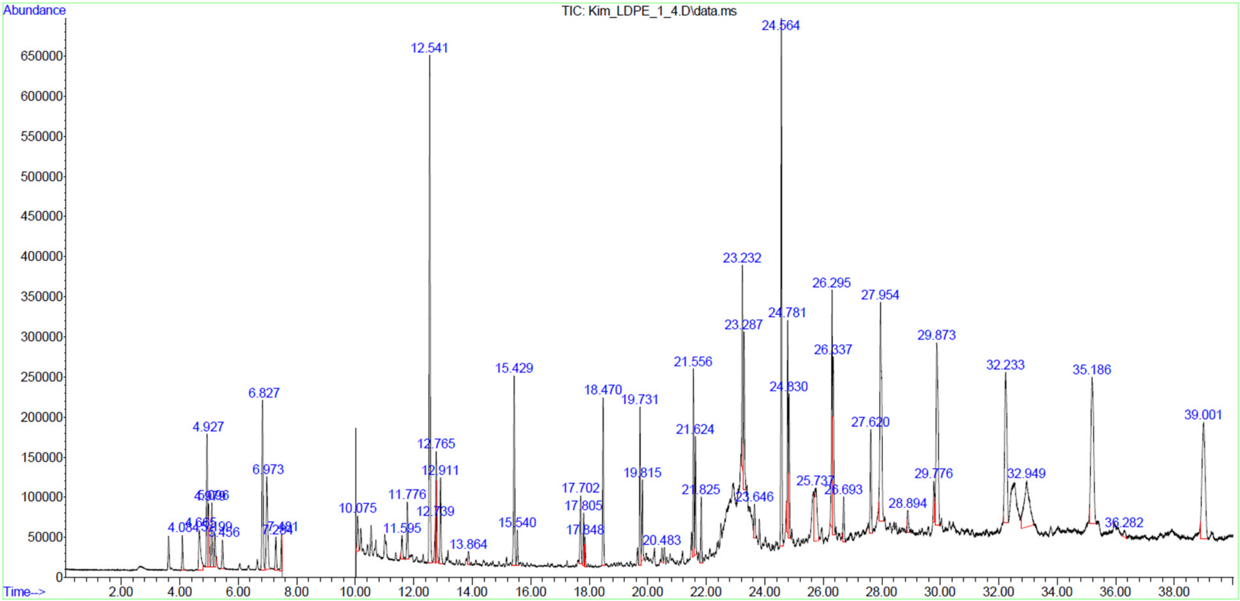

(f)

Figure S3. (a) GC-MS chromatogram of oil produced during catalytic pyrolysis of Fe(III) modified kaolin with PP with rate 1:1 (b) GC-MS chromatogram of oil produced during catalytic pyrolysis of Fe(III) modified kaolin with PP with rate 1:2 (c) GC-MS chromatogram of oil produced during catalytic pyrolysis of Fe(III) modified kaolin with PP with rate 1:4 (d) GC-MS chromatogram of oil produced during catalytic pyrolysis of Fe(III) modified kaolin with LDPE with rate 1:1 (e) GC-MS chromatogram of oil produced during catalytic pyrolysis of Fe(III) modified kaolin with LDPE with rate 1:2 (f) GC-MS chromatogram of oil produced during catalytic pyrolysis of Fe(III) modified kaolin with LDPE with rate 1:4.
